# An Explainable Multimodal Artificial Intelligence Model Integrating Histopathological Microenvironment and EHR Phenotypes for Germline Genetic Testing in Breast Cancer

**DOI:** 10.1002/advs.202502833

**Published:** 2025-05-29

**Authors:** Zijian Yang, Changyuan Guo, Jiayi Li, Yalun Li, Lei Zhong, Pengming Pu, Tongxuan Shang, Lin Cong, Yongxin Zhou, Guangdong Qiao, Ziqi Jia, Hengyi Xu, Heng Cao, Yansong Huang, Tianyi Liu, Jian Liang, Jiang Wu, Dongxu Ma, Yuchen Liu, Ruijie Zhou, Xiang Wang, Jianming Ying, Meng Zhou, Jiaqi Liu

**Affiliations:** ^1^ Institute of Genomic Medicine School of Biomedical Engineering Wenzhou Medical University Wenzhou 325027 China; ^2^ Department of Pathology National Cancer Center/National Clinical Research Center for Cancer/Cancer Hospital Chinese Academy of Medical Sciences and Peking Union Medical College Beijing 100021 China; ^3^ Department of Breast Surgery Chinese Academy of Medical Science and Peking Union Medical College Hospital Beijing 100730 China; ^4^ Department of Breast Surgical Oncology National Cancer Center/National Clinical Research Center for Cancer/Cancer Hospital Chinese Academy of Medical Sciences and Peking Union Medical College Beijing 100021 China; ^5^ School of Clinical Medicine Chinese Academy of Medical Sciences and Peking Union Medical College Beijing 100005 China; ^6^ Department of Breast Surgery the Affiliated Yantai Yuhuangding Hospital of Qingdao University Yantai 264000 China; ^7^ Department of Breast Surgery Sixth Affiliated Hospital of Harbin Medical University Harbin 150023 China; ^8^ State Key Laboratory of Molecular Oncology National Cancer Center/National Clinical Research Center for Cancer/Cancer Hospital Chinese Academy of Medical Sciences and Peking Union Medical College Beijing 100021 China; ^9^ Department of Pathology Second Affiliated Hospital of Harbin Medical University Harbin 150086 China; ^10^ Department of Breast Surgery Second Affiliated Hospital of Harbin Medical University Harbin 150086 China

**Keywords:** *BRCA1/2*, breast cancer, deep learning, electronic health records, tumor microenvironment, whole‐slide image

## Abstract

Genetic testing for pathogenic germline variants is critical for the personalized management of high‐risk breast cancers, guiding targeted therapies and cascade testing for at‐risk families. In this study, MAIGGT (Multimodal Artificial Intelligence Germline Genetic Testing) is proposed, a deep learning framework that integrates histopathological microenvironment features from whole‐slide images with clinical phenotypes from electronic health records for precise prescreening of germline *BRCA1/2* mutations. Leveraging a multi‐scale Transformer‐based deep generative architecture, MAIGGT employs a cross‐modal latent representation unification mechanism to capture complementary biological insights from multimodal data. MAIGGT is rigorously validated across three independent cohorts and demonstrated robust performance with areas under receiver operating characteristic curves of 0.925 (95% CI 0.868 – 0.982), 0.845 (95% CI 0.779 – 0.911), and 0.833 (0.788 – 0.878), outperforming single‐modality models. Mechanistic interpretability analyses revealed that *BRCA1/2*‐mutated associated tumors may exhibit distinct microenvironment patterns, including increased inflammatory cell infiltration, stromal proliferation and necrosis, and nuclear heterogeneity. By bridging digital pathology with clinical phenotypes, MAIGGT establishes a new paradigm for cost‐effective, scalable, and biologically interpretable prescreening of hereditary breast cancer, with the potential to significantly improve the accessibility of genetic testing in routine clinical practice.

## Graphical abstract

This study presents an explainable multimodal AI model, MAIGGT (Multimodal Artificial Intelligence Germline Genetic Testing), that combines whole‐slide histopathology with clinical data for accurate germline *BRCA1/2* mutation prescreening. By integrating digital pathology and EHR phenotypes, MAIGCT enables cost‐effective, scalable hereditary breast cancer risk assessment, improving genetic testing accessibility in clinical practice.

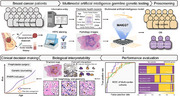

## 1 Introduction

Breast cancer is one of the most prevalent malignancies in women worldwide, with 5–10% of all cases attributable to germline pathogenic variants in cancer susceptibility genes.^[^ ^1^ ^]^ In particular, pathogenic variations in the highly penetrant genes *BRCA1* (17q21) [OMIM ID: 113705] or *BRCA2* (13q12–13) [OMIM ID: 600185] account for ≈15– 20% of familial breast cancer cases.^[^ ^1^, ^2^ ^]^ These germline mutations are associated with highly aggressive phenotypes, including an increased risk of ovarian and contralateral breast cancer and higher tumor grades.^[^ ^3^ ^]^ Moreover, *BRCA1/2*‐related breast cancers are particularly responsive to platinum and poly (ADP‐ribose) polymerase (PARP) inhibitors.^[^ ^4^ ^]^ Thus, identifying germline *BRCA1/2* mutations is critical for precise treatment, genetic counselling,^[^ ^5^ ^]^ and the optimization of health care costs.^[^ ^6^ ^]^ Since the first recommendation for germline *BRCA1/2* mutation testing in clinical guidelines in 2011,^[^ ^1^ ^]^ subsequent guidelines have broadened testing criteria to include patients regardless of age at diagnosis or family history.^[^ ^7^ ^]^ Clinical trials have further highlighted the importance of more effective screening methods for *BRCA1/2* mutations across different breast cancer phenotypes.^[^ ^8^ ^]^ Despite being one of the most commonly used criteria sets for prioritizing *BRCA1/2* genetic testing, current National Comprehensive Cancer Network criteria fail to identify nearly half of breast cancer patients with a clinically actionable germline *BRCA1/2* mutation.^[^ ^9^ ^]^

Previous studies have attempted to predict *BRCA1/2* mutation carriers based on clinical information, such as age, gender and family history of cancer, but achieved limited predictive performance.^[^ ^10^ ^]^ Recent efforts to incorporate detailed pathological features into phenotype‐driven prediction models have demonstrated higher accuracy in Asian populations.^[^ ^3^, ^10^ ^]^ This improved performance has shifted the focus of screening methods to pathological image‐based models, particularly given the well‐established genotype‐phenotype correlations in breast cancer. Tumors with specific genetic mutations often exhibit distinct pathological characteristics and corresponding image features.^[^ ^11^ ^]^ Whole‐slide images (WSIs) of breast cancer tissue provide a high‐resolution, detailed representation of the cell composition within the tumor microenvironment (TME), allowing for the quantitative characterization of cell‐level intratumoral heterogeneity (ITH).^[^ ^12^ ^]^ Recent advances in artificial intelligence (AI), particularly deep learning algorithms, have introduced innovative methods for analyzing pathological images.^[^ ^13^ ^]^ This technological advancement is further exemplified by Bilal et al.’s CAIMAN system, which demonstrated that AI‐based prescreening can achieve 99% sensitivity in identifying normal colorectal biopsies, significantly reducing pathologists workload while maintaining diagnostic accuracy for abnormal cases.^[^ ^14^ ^]^ These developments collectively highlight the show promise for predicting prognosis and therapeutic response related to breast pathology.^[^ ^15^ ^]^

However, the clinical application of image‐based AI for genotypic prediction remains underexplored. First, given that somatic mutations are more strongly associated than germline mutations to the tumor in genotype‐phenotype correlations, most image‐based AI models have been designed to predict somatic mutations.^[^ ^16^ ^]^ Second, previous models attempting to predict germline mutations from pathology images were trained on limited sample sizes without external validation and achieved inferior performance.^[^ ^17^ ^]^ Third, the biological significance of WSI features in image‐based AI models and their complementarity to clinical characteristics is largely unknown. On the other hand, current predictions of *BRCA1/2* mutation carriers are often based on single‐modal data, while the exploration of multi‐modal feature representation, such as aligning pathological images with phenotypic data to optimize clinical decision‐making and meet real‐world clinical applicability, remains insufficient.

Here, we developed MAIGGT (Multimodal Artificial Intelligence for Germline Genetic Testing), a novel deep learning framework that synergistically integrates histopathological features from WSIs with clinical phenotypes from electronic health records (EHRs) to accurately predict pathogenic *BRCA1/2* mutations. Through rigorous validation across multiple independent cohorts, we demonstrate that MAIGGT not only achieves state‐of‐the‐art predictive performance but also provides novel insights into the spatial biology of *BRCA1/2*‐associated tumors, offering a transformative approach to precision cancer risk assessment.

## 2 Results

### 2.1 Study Overview and Patient Characteristics

The MAIGGT framework initially incorporates a pathology image‐based model, WISE‐BRCA (Whole‐slide Images Systematically Extrapolate *BRCA1/2* mutations), which extracts high‐level histopathological feature representations from WSIs and predicts *BRCA1/2* mutation status using a multi‐scale transformer architecture. We validated the predictive performance of WISE‐BRCA through extensive multi‐center evaluation and interpretability analyzes. We then developed MAIGGT, which integrates histopathological microenvironment features from WSIs with clinical phenotypes from EHRs for precise multimodal joint prediction and assessed the complementarity between modalities.

**Figure 1:**
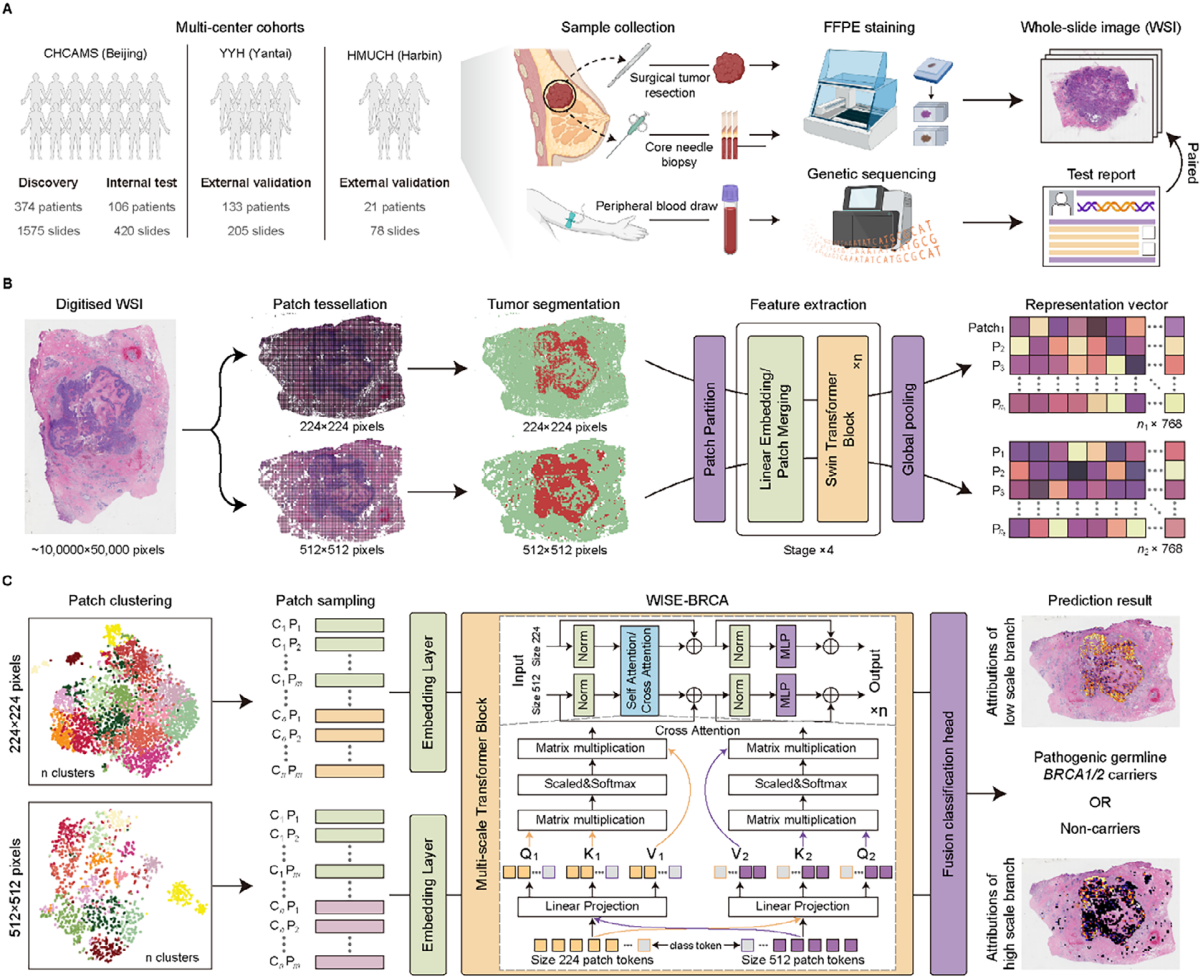
Study design and workflow of WISE‐BRCA. A) Multi‐center patient recruitment and sampling. Blood sampling, genetic sequencing, and pathogenic slide staining have done retrospectively. B) WSI pre‐processing and extraction of representative features. C) WSI sampling and schematic workflow of WISE‐BRCA. Abbreviations: WISE‐BRCA, Whole‐slide Images Systematically Extrapolate *BRCA1/2* mutations; EHR, electronic health record; FFPE, formalin‐fixed, paraffin‐embedded; CHCAMS, Cancer Hospital, Chinese Academy of Medical Sciences; YYH, Yantai Yuhuangding Hospital; HMUCH, Harbin Medical University Clinical Hospitals.

We collected 2278 H&E‐stained WSIs from 634 untreated breast cancer patients with matched clinical features across three Chinese hospitals: Cancer Hospital of the Chinese Academy of Medical Sciences in Beijing (CHCAMS), Yantai Yuhuangding Hospital in Shandong Province (YYH), and Harbin Medical University Clinical Hospitals in Heilongjiang Province (HMUCH) (**Figure** **1**A; Figure S1, Supporting Information). Baseline patient characteristics for each cohort are listed in **Table** **1**. After matching, 92/374 (24.6%) and 27/106 (25.5%) patients with germline *BRCA1/2* mutations were in the CHCAMS discovery set and internal test set, respectively. Germline *BRCA1/2* mutation carriers and non‐carriers had similar ages of onset, tumor sizes, lymph node statuses, and hormone receptors (HR) and human epidermal growth factor receptor 2 (HER2) status in the CHCAMS cohort (all *p* > 0.05; Figure S2, Supporting Information). However, carriers showed higher rates of family and personal history of breast and ovarian cancer, as well as a higher phenotype‐based risk score for *BRCA1/2* mutations (DrABC score)^[^ ^10^ ^]^ compared to non‐carriers (all P<0.05; Figure S2, Supporting Information). For the external validation cohorts, 30/133 (22.6%) and 10/21 (47.6%) patients were classified as *BRCA1/2* mutation carriers in the YYH and HMUCH cohorts, respectively.

**Table 1:** Baseline characteristics of patients enrolled in this study.

Characteristics	CHCAMS	YYH	HMUCH
No. of patients	480	133	21
No. of WSIs	1995	205	78
Age at diagnosis (Mean ± SD)	41.2 ± 9.3	46.5 ± 9.7	53.6 ± 9.8
Germline *BRCA1/2* mutation status			
*BRCA1* mutation carrier	65 (13.5%)	14 (10.5%)	9 (42.9%)
*BRCA2* mutation carrier	58 (12.1%)	16 (12.0%)	4 (19.0%)
Non‐carrier	361 (75.2%)	103 (77.4%)	11 (52.4%)
Personal history			
Any cancer	32 (6.7%)	19 (14.3%)	3 (14.3%)
Breast cancer	19 (4.0%)	9 (6.8%)	2 (9.5%)
Ovarian cancer	3 (0.6%)	2 (1.5%)	1 (4.8%)
Family history			
Any cancer	214 (44.6%)	32 (24.0%)	7 (33.3%)
Breast cancer	110 (22.9%)	11 (8.3%)	6 (28.6%)
Ovarian cancer	18 (3.8%)	1 (0.8%)	1 (4.8%)
Pancreatic cancer	12 (2.5%)	0 (0.0%)	1 (4.8%)
Male breast cancer	2 (0.4%)	0 (0.0%)	0 (0.0%)
Tumor size			
≤2cm	232 (48.3%)	59 (44.4%)	9 (42.9%)
>2cm	184 (38.3%)	60 (45.1%)	12 (57.1%)
Grade			
I	19 (4.0%)	11 (8.3%)	0 (0.0%)
II	183 (38.1%)	58 (43.6%)	9 (42.9%)
III	206 (42.9%)	41 (30.8)	12 (57.1%)
Estrogen receptor			
Positive	176 (36.7%)	76 (57.1%)	6 (28.6%)
Negative	252 (52.5%)	57 (42.9%)	15 (71.4%)
Progesterone receptor			
Positive	171 (35.6%)	82 (61.7%)	7 (33.3%)
Negative	252 (53.5%)	51 (38.3%)	14 (66.7%)
Ki67			
≤30%	193 (40.2%)	57 (42.9%)	9 (42.9%)
>30%	235 (49.0%)	76 (57.1%)	12 (57.1)
HER2			
Positive	36 (7.5%)	15 (11.3%)	1 (4.8%)
Negative	335 (69.8%)	118 (88.7%)	20 (95.2%)
Lymph nodes status			
Positive	209 (43.5%)	81 (60.9%)	15 (71.4%)
Negative	205 (42.7%)	39 (29.3%)	6 (28.6%)

Abbreviations: CHCAMS, Cancer Hospital, Chinese Academy of Medical Sciences; YYH, Yantai Yuhuangding Hospital; HMUCH, Harbin Medical University Clinical Hospitals; WSI, whole slide image; SD, standard deviation; HER2, human epidermal growth factor receptor 2.

### 2.2 Predicting Germline BRCA1/2 Mutation from Histology Images Using WISE‐BRCA

We developed a multi‐scale Transformer‐based deep learning model, WISE‐BRCA, to predict pathogenic germline *BRCA1/2* mutation carriers from H&E‐stained WSIs. While developing WISE‐BRCA, we initially evaluated the performance of the tumor segmentation model on the BCSS test set. The results show that the tumor segmentation model has a superior performance with an AUC of 0.894 (95% CI 0.882 – 0.906) for 224 × 224 pixel patches and 0.887 (95% CI 0.858 – 0.916) for 512 × 512 pixel patches (Figure S3, Supporting Information).

**Figure 2:**
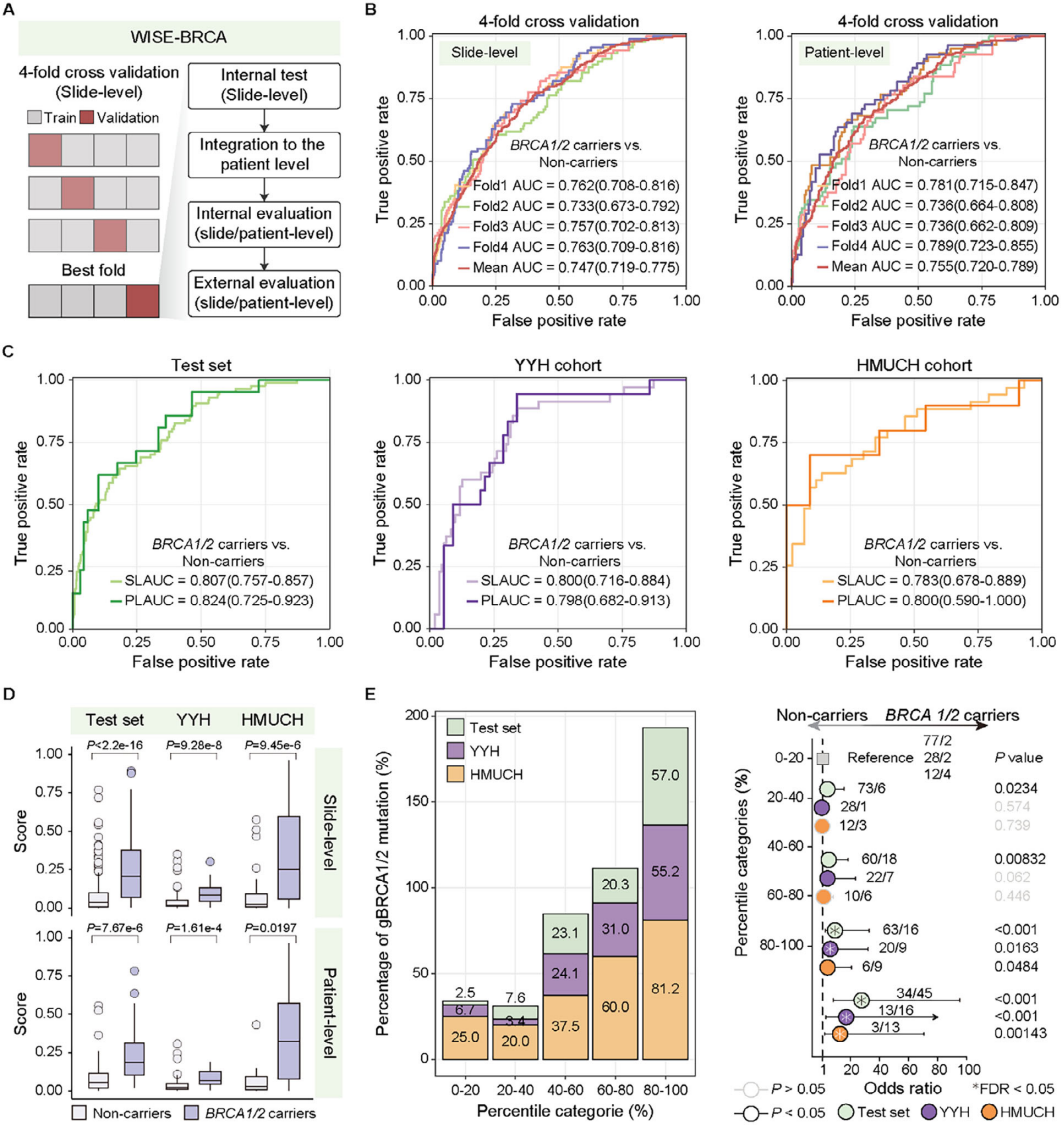
WISE‐BRCA's predictive power for germline *BRCA1/2* mutations. A) Model development and evaluation design. B) Slide‐level and patient‐level prediction efficacy of four‐fold cross‐validation in the discovery set (*n* = 358). C) Slide‐ and patient‐level prediction efficacy in the CHCAMS test set (*n* = 90) and YYH (*n* = 74) and HMUCH (*n* = 21) cohorts. D) Slide‐ and patient‐level prediction scores of the CHCAMS test set and YYH and HMUCH cohorts. E) Proportion of germline *BRCA1/2* mutation carriers across different model prediction score percentiles and odds ratios for associations between model prediction scores and germline *BRCA1/2* mutation status. Abbreviations: WISE‐BRCA, Whole‐slide Images Systematically Extrapolate *BRCA1/2* mutations; CHCAMS, Cancer Hospital, Chinese Academy of Medical Sciences; YYH, Yantai Yuhuangding Hospital; HMUCH, Harbin Medical University Clinical Hospitals.

For the development and evaluation of WISE‐BRCA, the CHCAMS cohort was split into a discovery set for model training and a test set for internal validation at the patient level in an 8:2 ratios (**Figure** **2**A). In the discovery set, WISE‐BRCA achieved a mean AUC of 0.747 (95% CI 0.719 – 0.775; 0.733 – 0.763 at the slide level) and a mean AUC of 0.755 (95% CI 0.720 – 0.789; 0.736 – 0.789 at the patient level) in the 4‐fold cross‐validation (Figure 2B). We further evaluated the performance of WISE‐BRCA in the CHCAMS test set and two external cohorts. WISE‐BRCA showed consistently high performance in predicting germline *BRCA1/2* mutation status, with AUCs of 0.807 (95% CI 0.757 – 0.857), 0.800 (95% CI 0.716 – 0.884), and 0.783 (95% CI 0.678 – 0.889) at the slide level and AUCs of 0.824 (95% CI 0.725 – 0.923), 0.798 (95% CI 0.682 – 0.913), and 0.800 (95% CI 0.590 – 1.000) at the patient level in the CHCAMS test set, YYH cohort, and HMUCH cohort, respectively (Figure 2C). The probabilities generated by WISE‐BRCA were significantly different between non‐carriers and *BRCA1/2* mutation carriers at both the slide and patient level (Figure 2D, *P* < 0.001). Furthermore, the proportion of *BRCA1/2* mutation carriers increased with higher prediction scores (Figure 2E). We also compared WISE‐BRCA with other H&E image‐based methods that are widely used in computational pathology, and achieved the best performance (Table S1, Supporting Information).

### 2.3 Model Reliability and Subgroup Analyses

**Figure 3:**
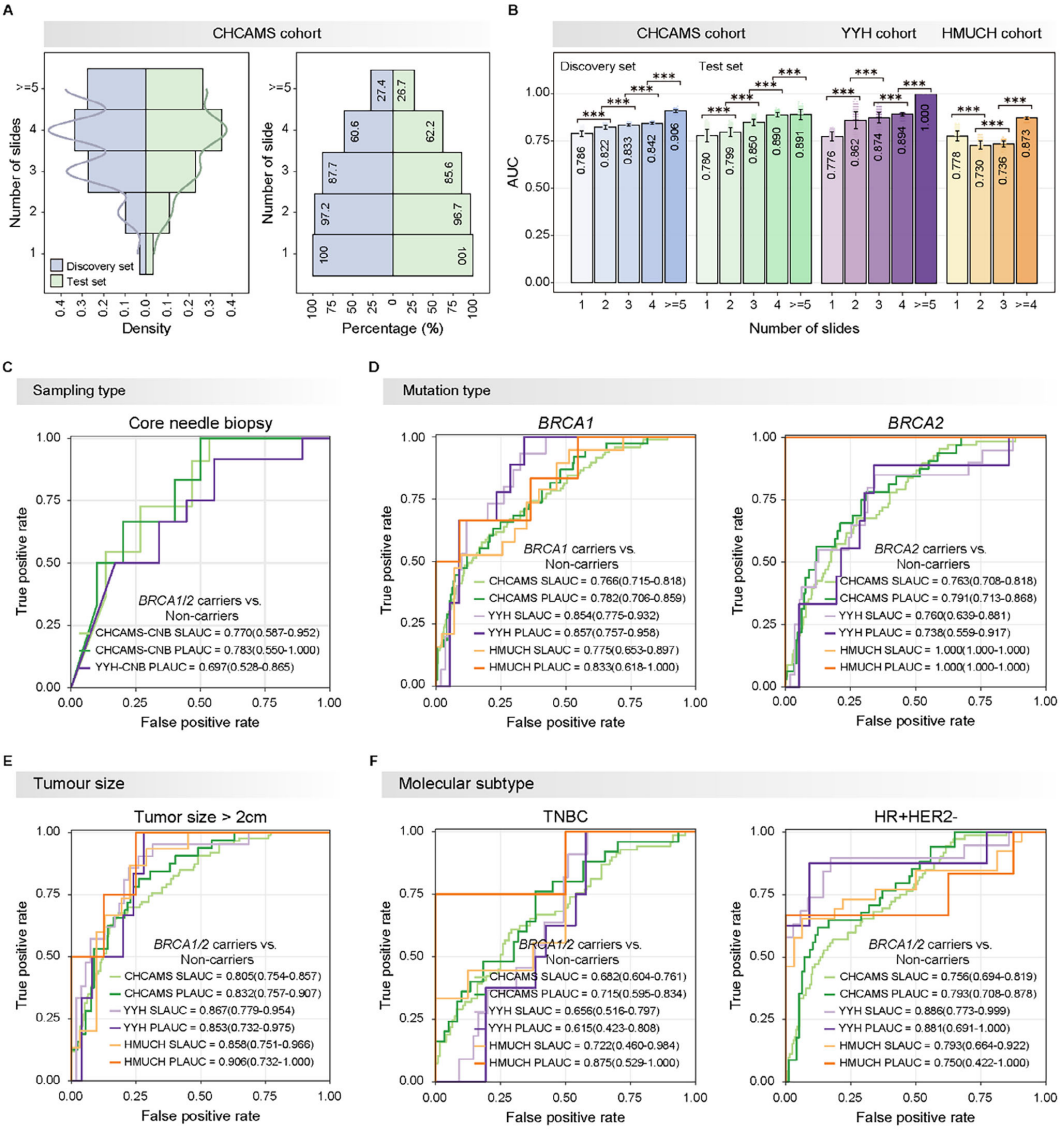
Model reliability and subgroup analyses. A) Distribution of the number of WSIs and percentage of patients with different numbers of WSIs in the CHCAMS cohort (Discovery set, *n* = 358; Test set, *n* = 90). B) Model prediction performance significantly improved as the number of slides for each patient increased from 1 to 5 or more (YYH cohort, *n* = 74; HMUCH cohort, *n* = 21). For a given number of slides *𝑛*, we sampled *𝑛* slides from each patient with at least *𝑛* slides and averaged the prediction scores of these *𝑛* slides to obtain the patient‐level prediction result. This sampling was performed 1000 times for each number of slides, and the numbers shown represent the average model performance across the 1000 samples. C) Prediction performance on core needle biopsy samples (CHCAMS‐CNB, *n* = 16; YYH‐CNB, *n* = 59). D) Prediction performance in *BRCA1* (CHCAMS, *n* = 247; YYH, *n* = 65; HMUCH, *n* = 17) or *BRCA2* (CHCAMS, *n* = 241; YYH, *n* = 65; HMUCH, *n* = 12) mutation carriers. E) Prediction performance in patients with different tumor sizes (CHCAMS, *n* = 124; YYH, *n* = 37; HMUCH, *n* = 12). F) Prediction performance in patients with different breast cancer molecular subtypes (TNBC: CHCAMS, *n* = 85; YYH, *n* = 34; HMUCH, *n* = 6; HR+HER2‐: CHCAMS, *n* = 131; YYH, *n* = 30; HMUCH, *n* = 14). Abbreviations: WISE‐BRCA, Whole‐slide Images Systematically Extrapolate germline *BRCA1/2* mutations; SL, slide‐level; PL, patient‐level; TNBC, triple‐negative breast cancer; HR, hormone receptor; HER2, human epidermal growth factor receptor 2; CHCAMS, Cancer Hospital, Chinese Academy of Medical Sciences; YYH, Yantai Yuhuangding Hospital; HMUCH, Harbin Medical University Clinical Hospitals.

To investigate the reliability and robustness of WISE‐BRCA, we first assessed model performance across patients with different numbers of slides in the three cohorts (**Figure** **3**A; Figure S4, Supporting Information). We observed significant improvements in model performance, as the number of slides per patient increased from 1 to ≥5, with AUCs increasing from 0.786 to 0.906 in the discovery set, from 0.780 to 0.891 in the test set, from 0.776 to 1.000 in the YYH cohort, and from 0.778 to 0.873 in the HMUCH cohort (Figure 3B).

We conducted further validation of WISE‐BRCA across various subgroups. WISE‐BRCA demonstrated acceptable performance on CNB samples, with a slide‐level AUC of 0.770 (95% CI 0.587 – 0.952) and patient‐level AUCs of 0.783 (95% CI 0.550 – 1.000) and 0.697 (95% CI 0.528 – 0.865) in the CHCAMS‐CNB and YYH‐CNB sets, respectively (Figure 3C). WISE‐BRCA maintained comparable efficacy between *BRCA1* and *BRCA2* mutation carriers, with slide‐level AUCs of 0.766 – 0.854 and 0.760 – 1.000, and patient‐level AUCs of 0.782 – 0.857 and 0.738 – 1.000 across the three cohorts (Figure 3D).

Notably, WISE‐BRCA achieved superior performance in patients with larger tumors (>2 cm), with slide‐level AUCs of 0.805 – 0.867 and patient‐level AUCs of 0.832 – 0.906 (Figure 3E). In the molecular subtype analysis, WISE‐BRCA shows lower AUCs in triple‐negative breast cancer (TNBC), with slide‐level AUCs of 0.656 – 0.722 and patient‐level AUCs of 0.615 – 0.875, demonstrating greater heterogeneity in the TNBC subtype^[^ ^18^ ^]^ compared to higher AUCs for the HR‐positive/HER2‐negative subtype (slide‐level: 0.756 – 0.886 and patient‐level: 0.750 – 0.881) (Figure 3F).

### 2.4 Morphological ITH of Tumor Nuclei and Immune Cell Infiltration

**Figure 4:**
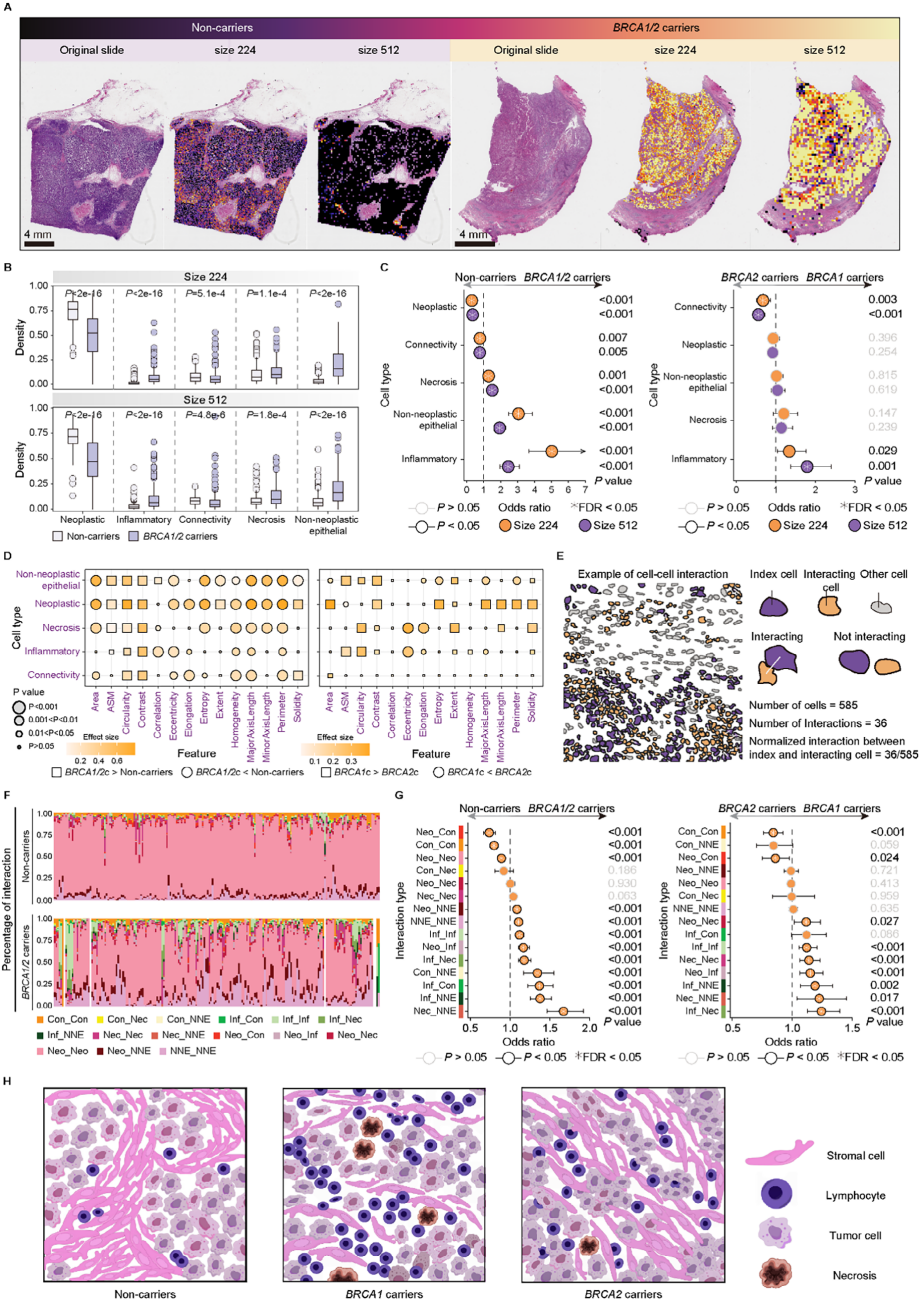
Biological interpretability for WISE‐BRCA and *BRCA1/2* mutation‐specific histomorphological patterns. A) Visualization of the gradient map from germline *BRCA1/2* mutation carriers and non‐carriers in patches of sizes 224 × 224 and 512 × 512 pixels. B) Densities of epithelial and TME cells in and *BRCA1/2* mutation carriers and non‐carriers. C) Odds ratios for associations between cell densities and germline *BRCA1/2* mutation status. D) Low‐scale geometric and textural features of cells in *BRCA1/2* mutation carriers and non‐carriers. E) Representative cell‐cell interaction metrics. F) High‐scale distribution of cell‐cell interactions in germline *BRCA1/2* mutation carriers and non‐carriers. G) Odds ratios for associations between cell‐cell interactions and *BRCA1/2* mutation status. H) Representation of genotype‐related TME features from representative patches showed enriched inflammatory cell infiltration and necrosis features in *BRCA1*‐mutated tumors, close cell connectivity in *BRCA2*‐mutated tumors, and stromal abundance and fewer lymphocytes in non‐carriers. Abbreviations: WISE‐BRCA, Whole‐slide Images Systematically Extrapolate *BRCA1/2* mutations. TME, tumor microenvironment.

To establish the interpretability of WISE‐BRCA and to identify *BRCA1/2* mutation‐specific histomorphological patterns, we used an Integrated Gradients (IG)‐based attribution method to calculate gradient values for patches of different sizes. Visualization of the gradient maps revealed distinct mutation‐related contextual features in the WSIs from *BRCA1/2* mutation carriers versus non‐carriers (**Figure** **4**A). We extracted and quantitatively analyzed representative patches critical for germline *BRCA1/2* prediction from 400 WSIs of non‐carriers and carriers in the CHCAMS cohort.

Based on genotype‐phenotype correlations, WISE‐BRCA facilitated biological interpretation in multiple dimensions: cell type, cell shape, and cell‐to‐cell interaction. *BRCA1/2* mutation carriers showed significantly higher enrichment of immune cells (*P* < 0.001) and non‐tumor stromal cells (*P *< 0.001) than non‐carriers. Additionally, immune cell density was significantly higher in *BRCA1* mutation carriers compared to *BRCA2* mutation carriers (*P* < 0.05) (Figure 4B,C; Figure S5, Supporting Information).

Low‐scale geometric and texture analysis revealed that non‐carrier cells had a large area, perimeter homogeneity, and small contrast, indicating relatively larger nuclei with less heterogeneity. Conversely, cells from *BRCA1/2* mutation carriers had a larger ASM and smaller area (*P* < 0.001), suggesting a closer cellular arrangement. Cells from *BRCA2* mutation carriers also showed greater eccentricity compared to *BRCA1* mutation carriers (*P* < 0.001), indicating more invasive growth patterns (Figure 4D; Figures S6, and S7, Supporting Information).

We further analyzed spatial cell‐cell interactions and observed distinct distributions of interactions between *BRCA1/2* mutation carriers and non‐carriers (Figure 4E). *BRCA1/2* mutation carriers, particularly *BRCA1*, show a significantly higher enrichment of immune cell‐related interactions (*P* < 0.001), which are marked by abundant immune cell infiltration and an inflammatory response (Figure 4F,G; Figures S6 and S7, Supporting Information). In contrast, non‐carriers showed less immune infiltration but more frequent stromal‐neoplastic cell interactions. These results indicate that *BRCA1/2* mutations may influence cell morphology and shape different TME. Representative patches reflecting these genotype‐related TME features are shown in Figure S8 (Supporting Information), with corresponding diagrams illustrating the enriched inflammatory infiltrates and necrosis in *BRCA1*‐mutated tumors, close tumor cell connectivity and more frequent mitosis in *BRCA2*‐mutated tumors, and stromal abundance with fewer lymphocytes in non‐carriers (Figure 4H).

### 2.5 Joint Prediction of *BRCA1/2* Mutation Carriers from Histology Images and Electronic Health Records Using MAIGGT

**Figure 5:**
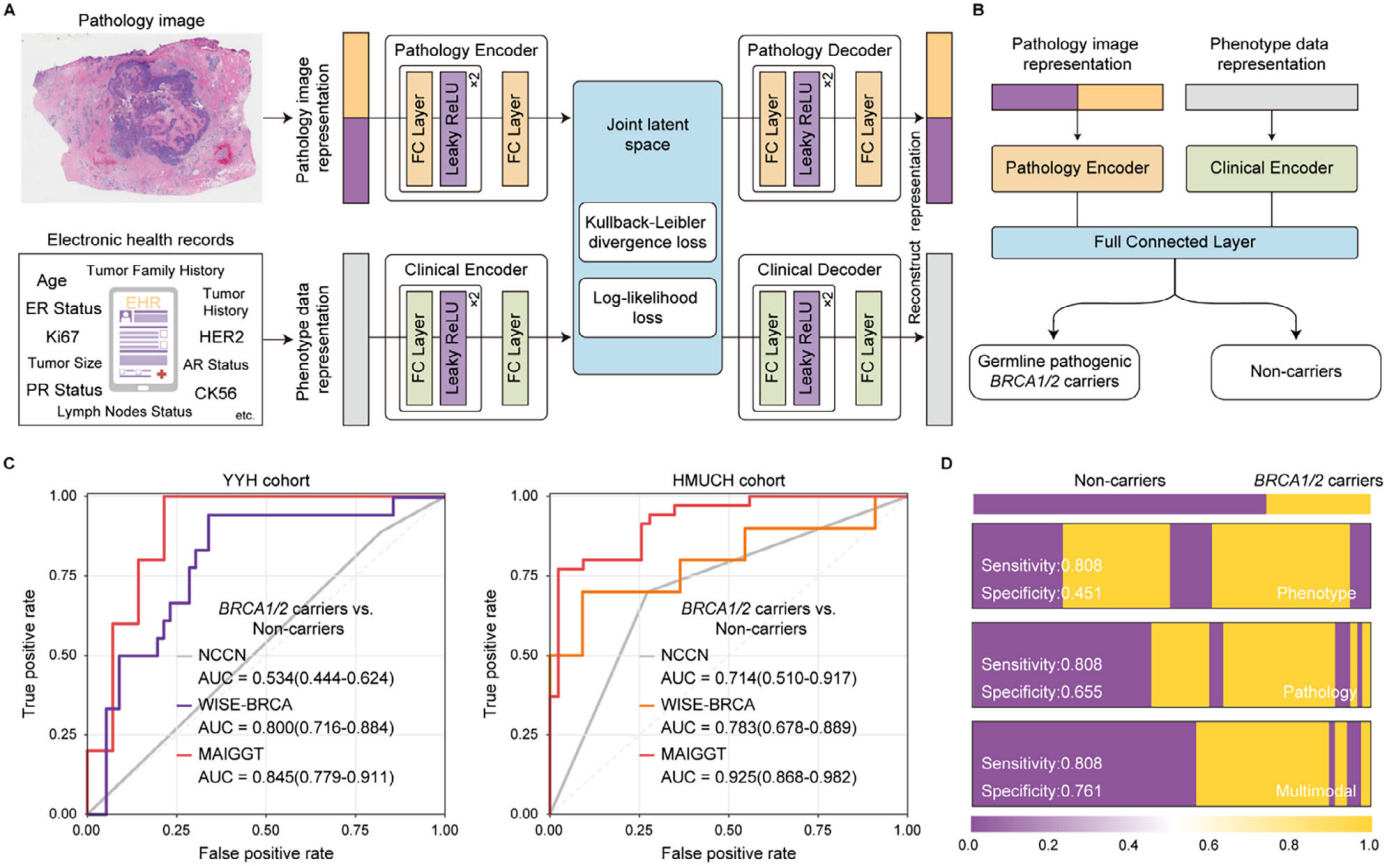
Efficacy of joint prediction of germline *BRCA1/2* mutation status from pathology images and clinical information. A) Development of the multi‐modal model MAIGGT. B) Joint prediction of germline *BRCA1/2* mutation status from pathology images and clinical information by MAIGGT. C) Patient‐level prediction efficacy in the YYH (*n* = 74) and HMUCH (*n* = 21) cohorts. D) MAIGGT achieved higher prediction accuracy than the other single‐modality models at 80% sensitivity. Abbreviations: MAIGGT, Multimodal Artificial Intelligence Germline Genetic Testing; EHR, electronic health record; ER, estrogen receptor; PR, progesterone receptor; HER2, human epidermal growth factor receptor 2; AR, androgen receptor; FC, full connected; CHCAMS, Cancer Hospital, Chinese Academy of Medical Sciences; YYH, Yantai Yuhuangding Hospital; HMUCH, Harbin Medical University Clinical Hospitals.

Building on WISE‐BRCA, the MAIGGT framework integrates learned high‐level histopathological feature representations with clinical phenotypes from EHRs (Table S2, Supporting Information) through a unified latent representation space (**Figure** **5**A,B). MAIGGT was initially developed using the CHCAMS cohort (Figure S9, Supporting Information) and then independently validated in two external cohorts. As a result, MAIGGT achieved AUCs of 0.845 (95% CI 0.779 – 0.911) and 0.925 (95% CI 0.868 – 0.982) in the YYH and HMUCH cohorts, respectively (Figure 5C), outperforming WISE‐BRCA, which relied solely on histology images for prediction. Finally, we assessed the relative contribution of individual features for the MAIGGT through SHAP, the overall SHAP values of the top‐10 pathology features and all phenotypic features are depicted in Figure S10 (Supporting Information). Among the phenotypic features, HER2 status, age at diagnosis, family history of tumor, and family history of breast cancer contributed the most significantly, and the pathological features derived from WISE‐BRCA demonstrated a consistently stable contribution to the model prediction (Figure S10, Supporting Information).

By incorporating clinical phenotypes, MAIGGT demonstrated superior prediction accuracy compared to other single‐modality models. At a sensitivity of 0.808, the multi‐modal MAIGGT achieved a specificity of 0.757, an improvement of 0.305 and 0.103 over the phenotype‐based and histology‐based WISE‐BRCA models, respectively (Figure 5C,D).

## 3 Discussion

In this study, we introduce MAIGGT, the first interpretable multi‐modal deep learning framework for predicting pathogenic germline *BRCA1/2* mutations in breast cancer patients using routine EHR and histology images. By synergistically integrating histopathological features from WSIs with clinical phenotypes from EHR through a unified latent representation space, MAIGGT achieves state‐of‐the‐art performance while providing novel biological insights into *BRCA1/2*‐associated tumor biology.

Pathological image‐based deep learning predictive models play an increasingly important role in many aspects of breast cancer management, including screening, diagnosis, staging (counting mitotic figures^[^ ^19^ ^]^ or identifying metastatic axillary lymph nodes^[^ ^20^ ^]^), biomarker evaluation (classifying molecular features^[^ ^21^ ^]^), prognostication, and therapeutic response prediction.^[^ ^15^ ^]^ According to our previous study, pathological features outperform clinical characteristics, such as age and family history,^[^ ^10^ ^]^ for predicting genetic mutations. Because patients with germline *BRCA1/2* mutations exhibit specific pathological features (e.g., absence of HR and HER2 expression^[^ ^5^, ^22^ ^]^), deep learning can help predict germline^[^ ^17a^ ^]^ or somatic^[^ ^16a^ ^]^ *BRCA1/2* mutations in breast and ovarian cancer^[^ ^3^, ^16^ ^]^ but is limited by small study cohorts,^[^ ^17a^ ^]^ lack of external validation,^[^ ^16a,c^ ^]^ and insufficient information on germline mutations.^[^ ^3^, ^16^ ^]^ Notably, the clinical utility of AI‐based prescreening has been independently validated in other cancer types. For example, Saillard et al. demonstrated that MSIntuit, a deep learning model for microsatellite instability (MSI) detection in colorectal cancer, achieved high sensitivity (96‐98%) across diverse cohorts and scanner platforms,^[^ ^23^ ^]^ highlighting the generalizability of such approaches. Our proposed WSI‐based WISE‐BRCA outperforms previous models due to its application to large populations of breast cancer patients, multi‐center external validation, germline mutation prediction, and innovative AI model construction. While WISE‐BRCA does not predict homogeneous recombination deficiency (HRD) status or somatic mutations, as achieved in some earlier studies,^[^ ^3^, ^24^ ^]^ its focus on germline *BRCA1/2* status is clinically relevant, particularly as an indicator for PARP inhibitor treatment.^[^ ^25^ ^]^ Moreover, HRD measurement in clinical practice often relies on germline *BRCA1/2* status. Despite the weaker association between germline mutations and phenotypic expression compared to somatic mutations, the strong predictive performance of WISE‐BRCA underscores the close relationship between germline mutations and pathological phenotypes, effectively capturing genotype information by analyzing detailed pathology image features.

WISE‐BRCA was developed using a transformer‐based architecture and a weakly supervised learning method that does not require costly manual annotation. Recognizing that WSIs at different magnifications reveal different levels of pathological features, WISE‐BRCA is designed with a multi‐scale architecture, and processes patches of different sizes to systematically analyze WSIs. WISE‐BRCA also introduces cross‐attention mechanisms to facilitate interactions between low‐scale morphological features and high‐scale, spatial‐oriented representations, resulting in a more synergistic and enriched histopathological feature representation. Based on the promising results achieved by pathology‐driven models, we aim to further integrate patient phenotypic data to explore potential performance improvements from multimodal information. Pathological images are high‐dimensional, complex, and unprocessed data, whereas clinical phenotypic information is low‐dimensional, manually refined and structured data that encapsulates human expertise. There are significant differences between the two in terms of dimensionality, data representation and information granularity. Considering that our MAIGGT uses a shared and unified deep generative modelling method to integrate the learned high‐level histopathological information from WISE‐BRCA with clinical phenotypic data, rather than simply fusing the raw data from the two modalities. Through multimodal prior distribution constraints and reconstruction, MAIGGT learns a joint common latent space between histopathological and phenotypic data, effectively aligning and integrating these modalities to extract intrinsic complementary features related to germline *BRCA1/2* mutations, thereby achieving superior predictive performance compared to single‐modality models and other integration methods (Table S3, Supporting Information).

Although achieving excellent performance, the interpretability of deep learning models remains a challenge, particularly in clinical settings where pathology is central to diagnosis. To address this barrier, we conducted a visualization analysis of regions of interest identified by WISE‐BRCA. The model revealed that *BRCA1* mutations were associated with higher inflammatory infiltration and necrosis, as well as off‐center nuclei and pushing tumor margins, while *BRCA2* mutations were associated with more active mitotic phases. In contrast, non‐carriers showed an abundance of stromal cells and tumor cells with centrally located nuclei. These results corroborate previous findings on the pathogenic mechanism of *BRCA1/2*, which indicate immune activation and nuclear morphological heterogeneity in *BRCA1/2*‐mutated breast cancers.^[^ ^26^ ^]^ Notably, although the use of HoverNet may introduce some bias, since our data are external datasets for HoverNet, any errors or biases introduced by HoverNet are generally uniformly distributed across the slides of both mutation and non‐mutation patients, without significant specificity. This may introduce bias into the numerical estimates within individual groups in our interpretability analysis, such as cell density estimates, but it will have little impact on the differences between groups that we primarily focus on, such as the differences in cell density between mutation carriers and non‐carriers. Moreover, clinical trials and drug development efforts suggest patients with *BRCA1/2* mutations may respond well to immunotherapy,^[^ ^27^ ^]^ and *BRCA2*‐mutated tumors are being investigated for potential resistance to CDK4/6 inhibitors because they regulate mitosis and the cell cycle.^[^ ^28^ ^]^ In this context, MAIGGT represents a more objective, feature‐naïve approach that innovatively adopts morphological classification and EHR phenotypes to systematically infer germline genotypes and their corresponding potential therapeutic targets.

Despite these promising findings, our study has several limitations. First, the retrospective nature of the study involving clinically matched patients introduces inherent selection bias and heterogeneity into the imaging data, which requires further validation in real‐world contexts. In addition, due to the clinical focus, we did not incorporate other genes or evaluate HRD status within DNA damage response pathways. However, MAIGGT is specifically designed to address clinical indications for intervention and to directly inform treatment selection. Consequently, the significant disparity between germline phenotypes and pathological features may lead to an underestimation of predictive results. Notably, our model demonstrated relatively lower performance in TNBC cases compared to hormone receptor‐positive subtypes. This may reflect the inherent biological heterogeneity of TNBC, where *BRCA1*‐associated tumors share histological features (e.g., immune infiltration, necrosis) with sporadic TNBCs, making discrimination difficult. In addition, the underrepresentation of TNBC cases (≈30% of our cohort) and the divergent molecular profiles of *BRCA1*‐ versus *BRCA2*‐associated tumors may contribute to this disparity. Future studies with expanded TNBC cohorts and subtype‐specific modeling approaches could further refine predictive accuracy in this clinically critical subgroup. Meanwhile, although MAIGGT demonstrates excellent predictive performance, it still lacks validation across different regions and ethnic groups. Therefore, larger, prospective, and cross‐ethnic multi‐center cohorts are needed to extend the application scenario of this promising deep learning model.

## 4 Conclusion

MAIGGT introduces a new paradigm for precision cancer risk assessment by demonstrating that deep learning can extract clinically relevant genetic insights from routine histopathology images. By bridging digital pathology with clinical genotypic data, this approach improves the accessibility, efficiency, and scalability of genetic testing. Ultimately, MAIGGT has the potential to transform personalized cancer prevention and treatment strategies, enabling early risk stratification, targeted interventions, and improved clinical decision‐making.

## 5 Experimental Section

### Study Design and Patient Cohorts

Participants from the CHCAMS cohort were sorted at patient‐level into a separate discovery set for model training and underwent four‐fold cross‐validation (*n* = 374), along with an internal test set for validation (*n* = 106). External validation was conducted independently in the YYH and HMUCH cohorts (*n* = 133 and 21, respectively) (Figure 1A). To avoid the potential influence of clinical characteristics and to alleviate data imbalance during model training, germline *BRCA1/2* mutation carriers and non‐carriers were matched from the CHCAMS and YYH cohorts in a 1: 2 – 3 ratio based on their age at diagnosis, tumor size, lymph node status, and molecular subtype. Participants were enrolled consecutively in the HMUCH cohort to mimic a real‐world scenario for breast cancer patients with high *BRCA1/2* mutation risk. Participants were excluded if they had received neoadjuvant chemotherapy, endocrine therapy, or radiotherapy prior to specimen collection. Treatment history was verified through electronic health records and pathological review to ensure that all samples analyzed represented treatment‐naïve tumor biology.

Additionally, the Breast Cancer Semantic Segmentation (BCSS) dataset,^[^ ^29^ ^]^ which includes WSIs from The Cancer Genome Atlas Breast Invasive Carcinoma cohort annotated by pathologists into six tissue regions, was incorporated. The BCSS dataset was split into a discovery set (80%) and test set (20%) for 5‐fold cross‐validation and testing of the tumor segmentation model as described later in this section.

The ethics committees of each participating institute approved the study (2021041314383902 for CHACMS, 2020–289 for YYH, LC2024‐052 for HMUCH). Written informed consent from patients was waived because of the complete anonymization of the study and the non‐identifying and retrospective nature of the collected data. The authors state that they have followed the principles outlined in the Declaration of Helsinki for all human experimental investigations.

### Genotype and Phenotypic Characteristics

Germline *BRCA1/2* mutations were previously analyzed through next‐generation sequencing of genomic DNA extracted from peripheral blood or saliva.^[^ ^10^ ^]^ The clinical significance of each mutation was evaluated according to guidelines of the American College of Medical Genetics and Genomics and the Association for Molecular Pathology through an in‐house pipeline.^[^ ^10^, ^30^ ^]^ Finally, the germline *BRCA1/2* mutation status of each participant was determined independently by two clinical geneticists.

Clinical and pathological data, including age at diagnosis, family and personal cancer history, pathological features, molecular subtype, and clinical stage, were extracted from each participant's EHR (Figure 1A). Molecular subtypes were defined based on the immunohistochemical status of hormone receptors, such as estrogen receptor (ER), progesterone receptor (PR), and HER2.

### Image Curation and Pre‐Processing

For patients with multiple slides, all available slides were retained to assess the effectiveness of the deep learning model at both the slide and patient level. In the CHCAMS and YYH cohorts, H&E‐stained slides were prepared from surgically resected and core needle biopsy (CNB) specimens using formalin‐fixed, paraffin‐embedded (FFPE) tissue blocks. These slides were digitized at 20× magnification using scanners (KF‐PRO‐040, FKBIO, China and 3D Panoramic SCAN SC150‐214705, DynaMax, China). In the HMUCH cohort, all H&E‐stained slides were prepared from surgical resection specimens and digitized at 40× magnification using the EasyScan One scanner (Motic, China). Poor‐quality slides were filtered out, including those with large areas of debris, folds, pen marks, blurred regions, missing diagnostic time information, poor visual quality, or abnormal staining. The filtering process was performed by two clinical pathologists who were blinded to patient identity, and in case of any disagreement, a third pathologist was involved for joint review.

To facilitate the analysis of gigapixel‐sized WSIs and capture information at different scales, WSIs meeting quality standards were tessellated into non‐overlapping patches of 224 × 224 and 512 × 512 pixels at 20× magnification. Patches with <50% tissue area were excluded from the analysis. To reduce staining variation and batch effects, patches from the external cohorts were subjected to staining normalization using a parameter‐variable staining normalization network.^[^ ^31^ ^]^ Similarly, WSIs from the BCSS dataset were tessellated into patches of different sizes, with patches containing >25% tumor area classified as tumor patches and the remainder as non‐tumor patches.

### Tumor Area Segmentation and Image Sampling

Emerging evidence indicates tumor areas are more closely associated with gene mutation compared to peri‐tumor and other non‐cancerous regions that may alter the tumor phenotype.^[^ ^32^ ^]^ Therefore, a tumor segmentation model was developed to automatically extract tumor areas from each WSI and to predict germline *BRCA1/2* mutations within these areas. The tumor segmentation model used ResNet50, pre‐trained on ImageNet, as the backbone and was trained on labelled patches of different sizes from the BCSS dataset for multi‐scale tumor area segmentation. Specifically, two tumor segmentation models were trained on patches of sizes 224 and 512, respectively. Both models used the cross‐entropy loss as an object function and were optimized using the AdamW optimizer with a batch size of 64, a learning rate of 7e‐4, and a weight decay of 5e‐4. Five‐fold cross‐validation was used to develop two models in the BCSS discovery set, with each fold trained for 100 epochs. The model with the highest AUC in the five‐fold cross‐validation was selected for further use.

To reduce computational complexity, a clustering‐based sampling strategy was implemented to extract patches with distinct histomorphological features from the tumor area (Figure 1B,C). CTransPath was used,^[^ ^33^ ^]^ a convolutional neural network and SwinTransformer‐based pathology foundation model, to generate 768‐dimensional feature representations for each patch. The K‐means algorithm was used to cluster patches into *N_c_* clusters, and *N_s_* patches were randomly sampled from each cluster, yielding a total of *N_c_* × *N_s_* patches per WSI (Figure S11, Supporting Information). Compared to random sampling, this clustering‐based sampling method comprehensively captures more complete information from WSIs and reduces the risk of missing significant patches with less common histomorphological features.

### Architecture of WISE‐BRCA

As shown in Figure 1C, WISE‐BRCA consists of three main components: embedding layer, multi‐scale Transformer block and fusion classification head. The embedding layer includes a linear layer, a batch normalization layer, and a ReLU activation function. In practical applications, WISE‐BRCA sets two embedding layers to embed the feature representations $Z_{l}=\left\{z_{1,1},\;z_{1,2},\;z_{1,3},\;\text{…},\;z_{c_{l},s_{l}}\right\}\;,\;z_{i,j}\in R^{d}$ and $Z_{h}=\left\{z_{1,1},\;z_{1,2},\;z_{1,3},\;\text{…},\;z_{c_{h},s_{h}}\right\}\;,\;z_{i,j}\in R^{d}$ of 224 × 224 an 512 × 512 sized patches from cluster sampling, with output embedding features 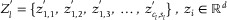 and 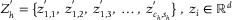, where *c_l_*, *s_l_* and *c_h_*, *s_h_* represent the number of clusters and the number of samples at different sampling sizes, respectively, and *d* represents the dimension of patch embeddings.

Then, *Z_l_* and *Z_h_* were treated as tokens and forwarded to twelve stacked multi‐scale Transformer blocks. Each block contains a low‐scale branch and a high‐scale branch, which process *Z_l_* and *Z_h_* respectively, to extract *BRCA1/2* mutation‐related multi‐scale information for prediction. Traditional self‐attention does not allow for cross‐scale information interaction and fusion due to the independence of branches. Therefore, WISE‐BRCA introduces cross‐attention to bridge the gap between low‐scale morphological features and high‐scale spatially oriented representations, allowing information interaction between the branches and generating systematic high‐level discriminative features. The cross‐attention would be used interchangeably with self‐attention in consecutive multi‐scale Transformer blocks. Suppose the *X_l_*, $X_{cls}^{l}$ $\in R^{c_{l}s_{l}\times d}$, and *X_h_*, $X_{cls}^{h}$ $\in R^{c_{h}s_{h}\times d}$ are the patch tokens and class tokens output by the previous multi‐scale Transformer block, the calculation process of the cross attention was as follows:

(1)

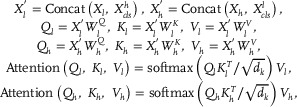

where *Q_l_*, *K_l_*, *V_l_* $\in R^{c_{l}s_{l}\times d_{k}}$ and *Q_h_*, *K_h_*, *V_h_* $\in R^{c_{h}s_{h}\times d_{k}}$, are the query, key and value matrices; $W_{l}^{Q}$, $W_{l}^{K}$, $W_{l}^{V}$ $\in R^{d\times d_{k}}$ and $W_{h}^{Q}$, $W_{h}^{K}$, $W_{h}^{V}$ $\in R^{d\times d_{k}}$ are learnable parameters. Multiple heads were also allocated in cross‐attention as follows:

(2)

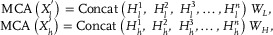

where $H_{l}^{i}=\;\mathrm{Attention}\left(Q_{l}^{i},\;K_{l}^{i},\;V_{l}^{i}\right)$ and $H_{h}^{i}=\;\mathrm{Attention}\left(Q_{h}^{i},\;K_{h}^{i},\;V_{h}^{i}\right)$ for i∈{1,2,…n}; $W_{L}\in R^{nd_{k}\times d}$ and $W_{H}\in R^{nd_{k}\times d}$ are learnable parameters; *n* represents the number of cross attention heads. Here, *c_l_*, *s_l_*, *c_h_*, *s_h_* and *n* were set to 30, 60, 30, 60, and 12, respectively. The dimension of *d* and *d_k_* were 768 and 64.

Finally, a systematic feature representation of 1536 dimensions was generated by concatenating the class tokens from the low‐scale and high‐scale branches. Meanwhile, this discriminative feature was forward to the linear fusion classification head to obtain the slide‐level *BRCA1/2* mutation prediction result.

During training, binary cross‐entropy was used with logits loss as the object function and AdamW as the optimizer. The initial learning rate was set to 1e‐5, weight decay was 1e‐3 and batch size was 32. WISE‐BRCA was trained using 4‐fold cross‐validation, with each fold trained for 150 epochs and early stopping after 20 epochs of no improvement. The model with the highest validation AUC among the 4‐fold cross‐validation was selected as the best model for internal test and external validation. To expedite model convergence, the GradualWarmupScheduler was used for a five epochs warm‐up, followed by CosineAnnealingLR with T‐max set to 95 epochs to adjust the learning rate.

### Attribution and Visualization of WISE‐BRCA Predictions

To ensure the intrinsic interpretability of WISE‐BRCA and reveal biological changes associated with *BRCA1/2* mutations from an AI perspective, model predictions were attributed using the Integrated Gradients (IG) method.^[^ ^34^ ^]^

For the patch feature representations *Z_l_* and *Z_h_* of the input WSI, IG calculates an importance‐related gradient value for each patch as follows:

(3)

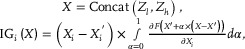

where *F*(*x*) is the WISE‐BRCA model; *X* and *X*′ represent the feature representations and baseline features of all input patches; *i* represents the *i*‐th patch feature representation in *X*.

After the computation, IG will output IG gradient values of the same size as the input. The IG gradient values were then averaged across patches at different scales as the gradient value for each patch. The IG gradient values were used to quantify the importance of each patch in the model's prediction, with larger gradient values indicating a stronger association with *BRCA1/2* mutations. For slide‐level visualisation, cluster sampling was performed 500 times per WSI, and predictions were made for each sampling. The IG gradients for each patch were averaged to obtain macro‐attribution results for the WSI. At the patch level, 200 WSIs were selected from germline *BRCA1/2* mutation carriers with the highest prediction probabilities, and 200 WSIs were selected from non‐carriers with the lowest prediction probabilities. The top 10 highest gradient value patches from germline *BRCA1/2* mutation carriers and the top 10 lowest gradient value patches from non‐carriers were then selected for morphological and spatial interaction analyses.

### Nuclei Segmentation and Feature Extraction

Nuclei segmentation was performed using HoVer‐Net^[^ ^35^ ^]^ and pre‐trained on the PanNuke annotated pan‐cancer pathology image dataset to classify nuclei into six types: neoplastic, inflammatory, connective, dead, non‐neoplastic stromal, and unlabeled (Figure S12, Supporting Information). Based on the centroid and contour of the nucleus within its corresponding patch, 14 total morphological and texture features were obtained to quantitatively characterize the properties of each nucleus (Table S4, Supporting Information).^[^ ^36^ ^]^

### Spatial Cell‐Cell Interaction Analysis

Cells were defined as interacting if their segmentation masks were in direct contact (i.e., the contour of the nuclei was connected); spatial interactions for all cells were calculated based on this criterion. Furthermore, cell type annotations were incorporated, which identified 15 cell‐cell interactions between different cell types. The abundance of each interaction type within a patch was calculated as the total number of interactions between two cell types divided by the total number of cells.

### Fine‐Tuning WISE‐BRCA on Fresh‐Frozen Samples

To improve the generalization of WISE‐BRCA, transfer learning was performed on fresh frozen (FF) samples. Specifically, the model was fine‐tuned, trained initially on FFPE samples, using the Finetune set for 50 epochs with an early stopping criterion of 10 epochs. Independent performance evaluations were then conducted on the CHCAMS‐CNB and YYH‐CNB cohorts. During the fine‐tuning, the binary cross‐entropy was used with logits loss as the object function and AdamW as the optimizer with a learning rate of 1e‐3, weight decay of 5e‐4 and a batch size of 2. The learning rate was initially adjusted using the GradualWarmupScheduler for the first 10 epochs, followed by CosineAnnealingLR with T‐max set to 20 epochs to further modulate the learning rate.

### Architecture of MAIGGT

Based on WISE‐BRCA, clinical information of patients (Table S2, Supporting Information) was incorporated to design a multi‐modal deep learning model, MAIGGT, for jointly predicting germline *BRCA1/2* mutations. An unsupervised deep generative multi‐channel variational auto‐encoder (mcVAE)^[^ ^37^ ^]^ was employed to learn a shared common latent space and generating unified representations for histopathological and phenotypic data. For the input pathological image representation *X_patho_* derived from WISE‐BRCA and standardized clinical information *X_pheno_* (The standardization method was the same as the previous study.),^[^ ^10^ ^]^ MAIGGT employs two pairs of encoders and decoders (Table S5, Supporting Information) for training. To optimize the Evidence Lower Bound (ELBO), achieving cross‐modal learning and reasoning capabilities, two loss functions *L_rec_* and *L_reg_* were delineated. Among them, *L_rec_* represents the reconstruction term for intra‐modal self‐reconstruction and inter‐modal cross‐reconstruction between pathological and phenotypic data. *L_reg_* is the regularization term that constrains the distributions of the latent variables for both modalities remain close to the prior distribution. The detailed description is as follows:

(4) $$\begin{array}{ccc} & & L_{rec}=E_{Z_{patho},\;Z_{pheno}}\left[\sum_{i,\;j}\log\;p\left(X_{i}|Z_{j}\right)\right]\\ & & L_{reg}=\beta \cdot \left(D_{KL}\left(q\left(Z_{patho}|X_{patho}\right)\left|\right|p\left(Z\right)\right)+D_{KL}\left(q\left(Z_{pheno}|X_{pheno}\right)\left|\right|p\left(Z\right)\right)\right) \end{array}$$

Here, $Z_{patho}∼\;q\left(Z_{patho}|X_{patho}\right)$ and $Z_{pheno}∼\;q\left(Z_{pheno}|X_{pheno}\right)$ are the posterior distributions of pathological and phenotypic features, respectively. p(Z)=N(0,I) represents the standard Gaussian distribution. β represents the weight coefficient of the constraint term. *i* and *j* represent the pathological or phenotypic channels. *D_KL_* represents the Kullback‐Leibler Divergence, which is formulated by

(5) $$D_{KL}\left(p||q\right)=\frac{1}{2}\;\sum_{k=1}^{K}\left(\sigma_{k}^{2}+\mu_{k}^{2}-1-\log\sigma_{k}^{2}\right)$$

where $p\;=N(\mu_{1},\sigma_{1}^{2})$, and $q\;=N(\mu_{2},\sigma_{2}^{2})$. Finally, the reconstruction and regularization losses were combined as the objective function L as follows:

(6) $$L=L_{reg}\;-L_{rec}$$

During training, the encoders of each channel generate the posterior distributions for the inputs of their respective modalities, while the decoders reconstruct the multimodal data from the reparameterized sample $Z_{i}=\mu_{i}+\varepsilon_{i}\cdot \sigma_{i}$ $\left(\varepsilon_{i}∼N\left(0,I\right)\right)$. The model was trained using AdamW optimizer with a batch size of 32, a learning rate of 5e‐4, and a weight decay of 5e‐4 for 2000 epochs, with an early stopping criterion set at 100 epochs. To expedite model convergence, the GradualWarmupScheduler was used for 50 epochs of warm‐up, followed by CosineAnnealingLR with T‐max of 50 epochs to adjust the learning rate.

After that, the learned unified latent representation of histopathological and phenotypic data was fed into a multi‐modal fusion classification head (Table S6, Supporting Information) for prediction and uses the binary cross‐entropy with logits loss as the object function. The multimodal fusion classification head was also optimized using the AdamW optimizer and trained for 100 epochs with a batch size of 60, a learning rate of 5e‐4, weight decay of 5e‐4, a dropout rate of 0.4, and an early stopping criterion set at 10 epochs.

### Hardware and Software

The PyTorch (version 1.13.1), Pandas (version 1.3.4), Numpy (version 1.24.4), OpenCV (version 4.5.3.56), histolab (version 0.5.1), and Feather (0.4.1) were used in this project. All deep learning models were implemented on two NVIDIA 4090 GPUs with CUDA11.7 and cuDNN 8.5.0.

### Statistical Analysis

Statistical analysis was performed using R (version 4.2.2) and Python (version 3.8). The unpaired t‐test was used to compare continuous variables with a normal distribution, while the Mann‐Whitney U test or Kruskal‐Wallis test was used for non‐normally distributed continuous variables. Means and standard deviations (SDs) summarized continuous variables, whereas categorical variables were expressed as percentages. The area under the curve (AUC)‐receiver operating characteristic was calculated using the pROC package (version 1.18.0). Odds ratios were calculated using univariate logistic regression. The 95% confidence intervals (CIs) were calculated using the non‐parametric bootstrap method. All tests were two‐tailed, with a significance level set at *P* < 0.05.

## Conflict of Interest

The authors declare no conflict of interest.

## Author Contributions

Z.J.Y., C.Y.G., J.Y.L., Y.L.L., and L.Z. contributed equally to the work. J.Q.L. and M.Z. conceived the idea and designed the study. J.Y.L., Y.L.L., L.Z., P.M.P., Y.X.Z., T.X.S. and L.C. collected and double‐checked the clinical data. Z.J.Y. and M.Z. developed the deep learning algorithm. Z.J.Y., JQ.L., J.Y.L., and C.Y.G. analyzed the results. C.Y.G., J.Y.L., Y.L.L., L.Z., and J.M.Y. analyzed the pathological images. Z.J.Y., J.Y.L., C.Y.G., J.Q.L., and M.Z. wrote and revised the manuscript. All the authors participated in revising the manuscript before submission and the formal revision, including literature searching, information extraction, and text modification.

## Supporting information



Supporting Information

## Data Availability

The datasets generated during and/or analyzed during the current study are available from the corresponding author on reasonable request. Codes used in this study are publicly available at https://github.com/ZhoulabCPH/MAIGGT/.
