# Supplementary material for: An Explainable Multimodal Artificial Intelligence Model Integrating Histopathological Microenvironment and EHR Phenotypes for Germline Genetic Testing in Breast Cancer
Source: Adv Sci (Weinh). 2025 May 29;12(31):e02833. doi: 10.1002/advs.202502833 (PMC12376594; doi:10.1002/advs.202502833)
Supplement: Supplementary file 1 — Supporting Information [file ADVS-12-e02833-s001.pdf]

ADVANCED SCIENCE
Open Access

Supporting Information

for *Adv. Sci.*, DOI 10.1002/advs.202502833

An Explainable Multimodal Artificial Intelligence Model Integrating Histopathological Microenvironment and EHR Phenotypes for Germline Genetic Testing in Breast Cancer

Zijian Yang, Changyuan Guo, Jiayi Li, Yalun Li, Lei Zhong, Pengming Pu, Tongxuan Shang, Lin Cong, Yongxin Zhou, Guangdong Qiao, Ziqi Jia, Hengyi Xu, Heng Cao, Yansong Huang, Tianyi Liu, Jian Liang, Jiang Wu, Dongxu Ma, Yuchen Liu, Ruijie Zhou, Xiang Wang, Jianming Ying, Meng Zhou* and Jiaqi Liu**

Supporting Information

An explainable multimodal artificial intelligence model integrating histopathological microenvironment and EHR phenotypes for germline genetic testing in breast cancer

Zijian Yang, Changyuan Guo, Jiayi Li, Yalun Li, Lei Zhong, Pengming Pu, Tongxuan Shang, Lin Cong, Yongxin Zhou, Guangdong Qiao, Ziqi Jia, Hengyi Xu, Heng Cao, Yansong Huang, Tianyi Liu, Jian Liang, Jiang Wu, Dongxu Ma, Yuchen Liu, Ruijie Zhou, Xiang Wang, Jianming Ying, Meng Zhou, Jiaqi Liu

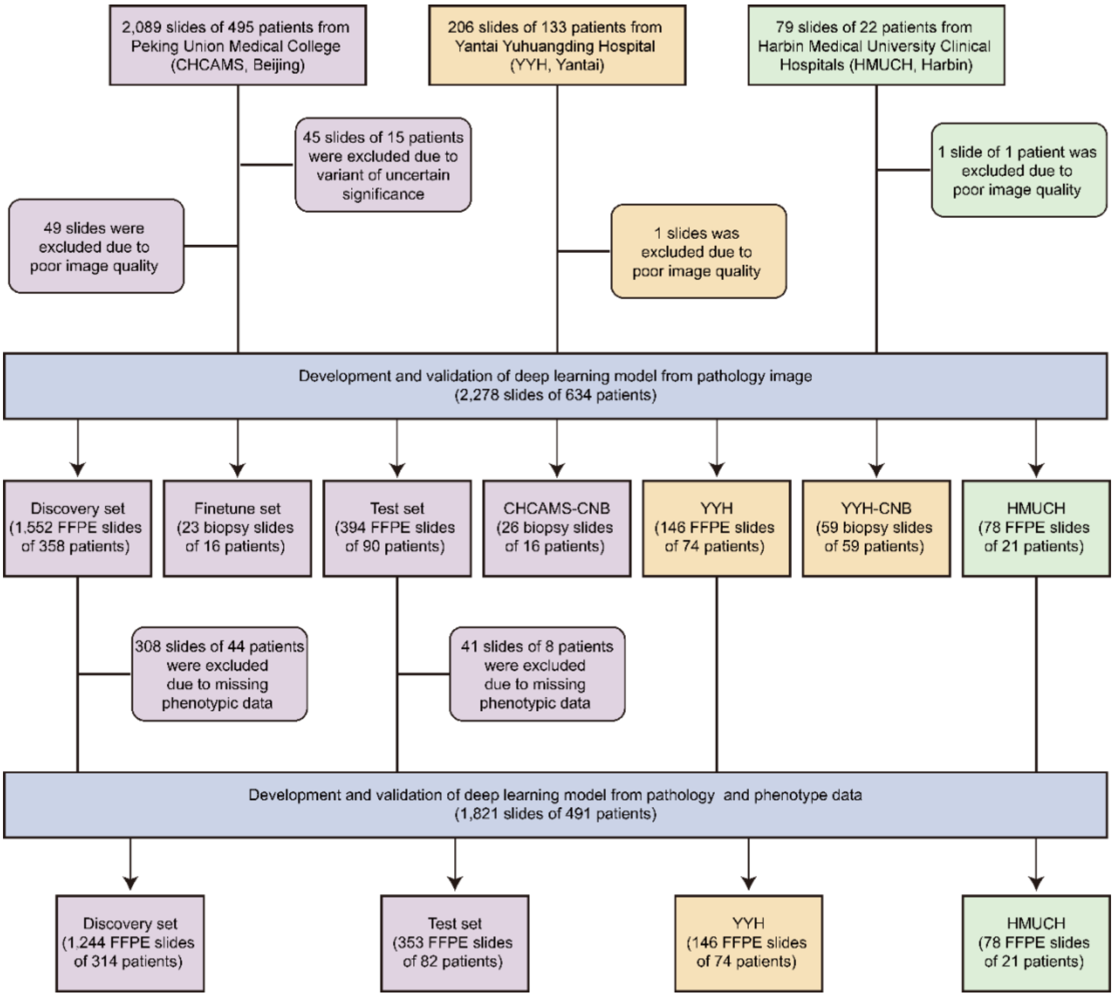

Figure S1. Multi-center patient and WSI recruitment.

Abbreviations: WSIs: whole slide images; FFPE: fixed paraffin-embedded; CHCAMS, Cancer Hospital, Chinese Academy of Medical Sciences; YYH, Yantai Yuhuangding Hospital; HMUCH, Harbin Medical University Clinical Hospitals.

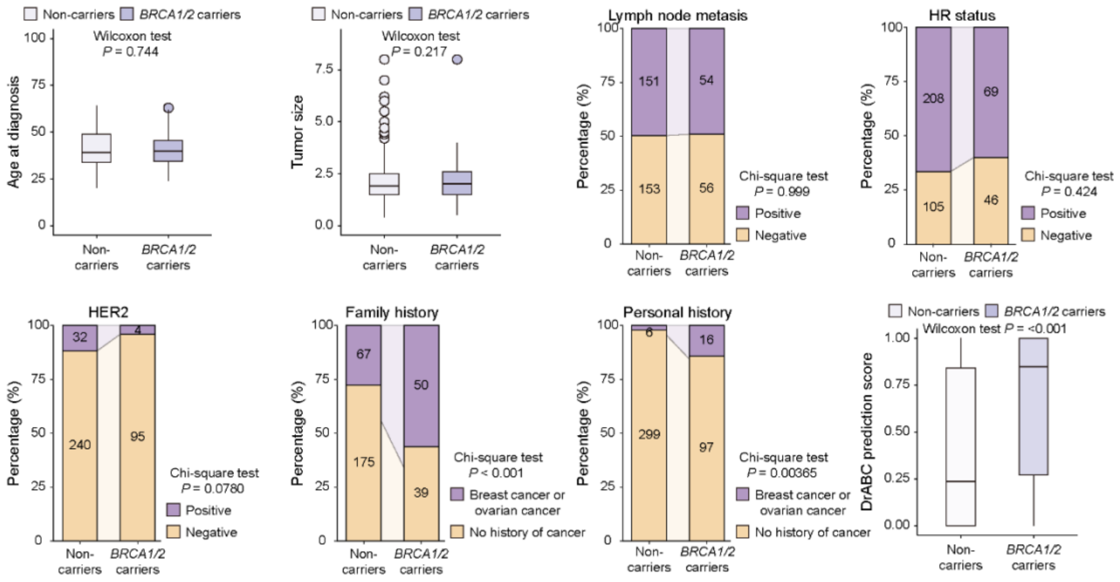

Figure S2. Comparison of clinicopathological characteristics between germline *BRCA1/2* mutation carriers and non-carriers in the CHCAMS cohort.

Abbreviations: HR: hormone receptor; HER2: human epidermal growth factor receptor 2; CHCAMS, Cancer Hospital, Chinese Academy of Medical Sciences.

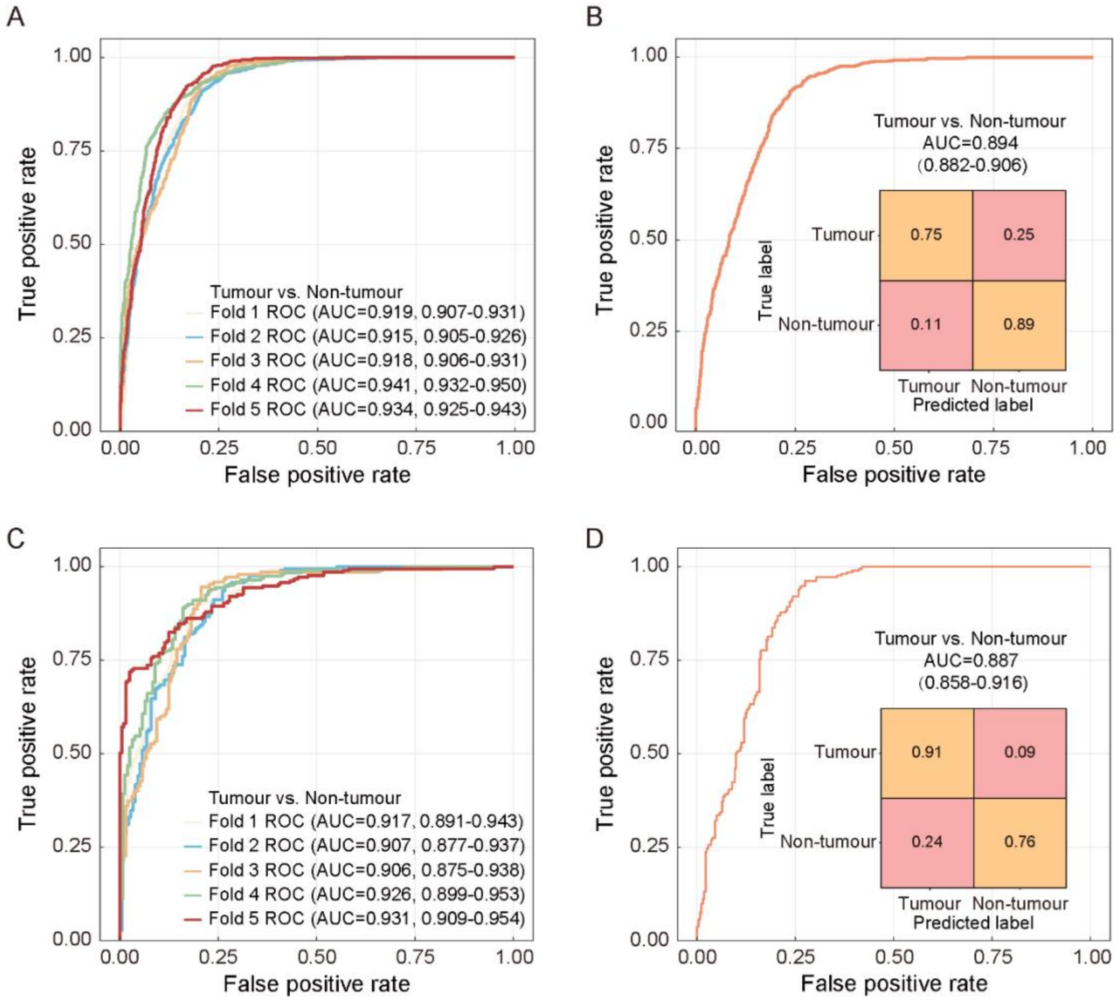

Figure S3. Prediction performance of the tumor segmentation model. Receiver operator curve (**A**) and confusion matrix (**B**) of 5-fold cross-validation in the BCSS discovery cohort for the tumor segmentation model trained on patches of size 224. Receiver operator curve **(C)** and confusion matrix (**D**) of testing in the BCSS test set for tumor segmentation model trained on patches of size 512.

Abbreviations: BCSS: Breast Cancer Semantic Segmentation.

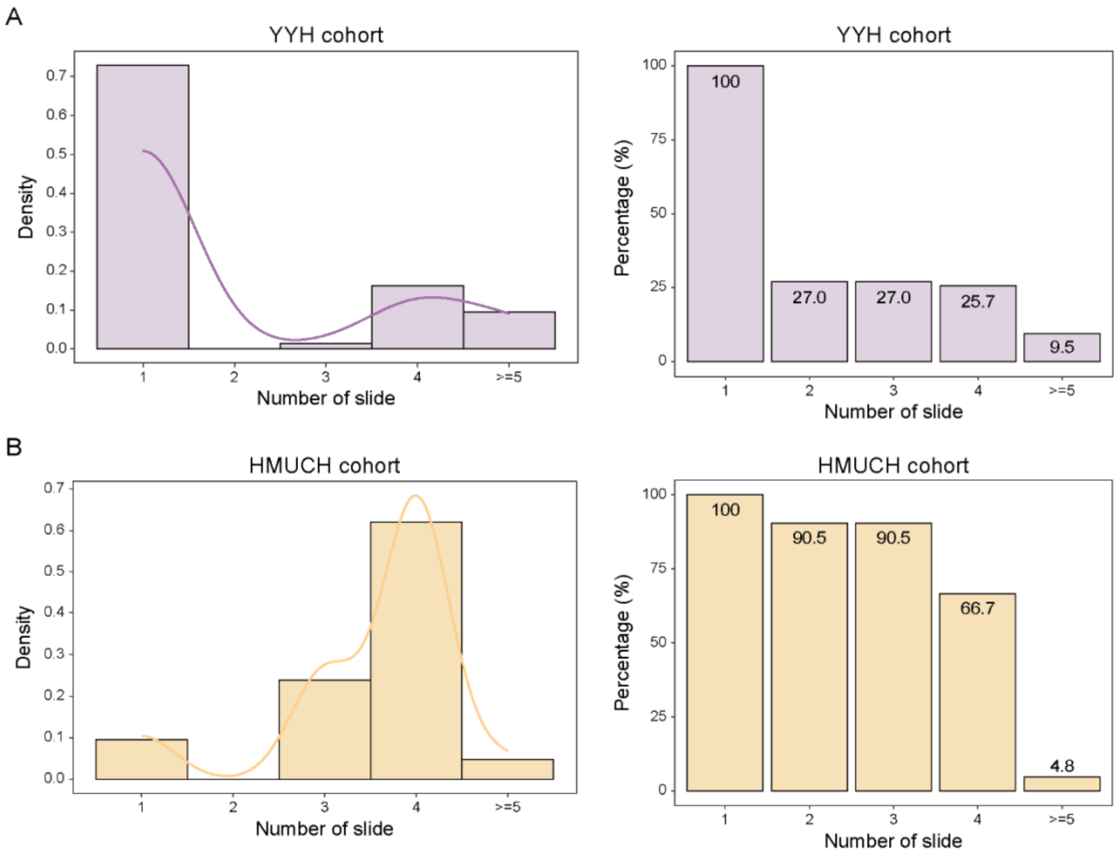

Figure S4. Distribution of the number of WSIs and the percentage of patients under different numbers of WSIs in the (A) YYH cohort and (B) HMUCH cohort.

Abbreviations: WSIs: whole slide images; YYH, Yantai Yuhuangding Hospital; HMUCH, Harbin Medical University Clinical Hospitals.

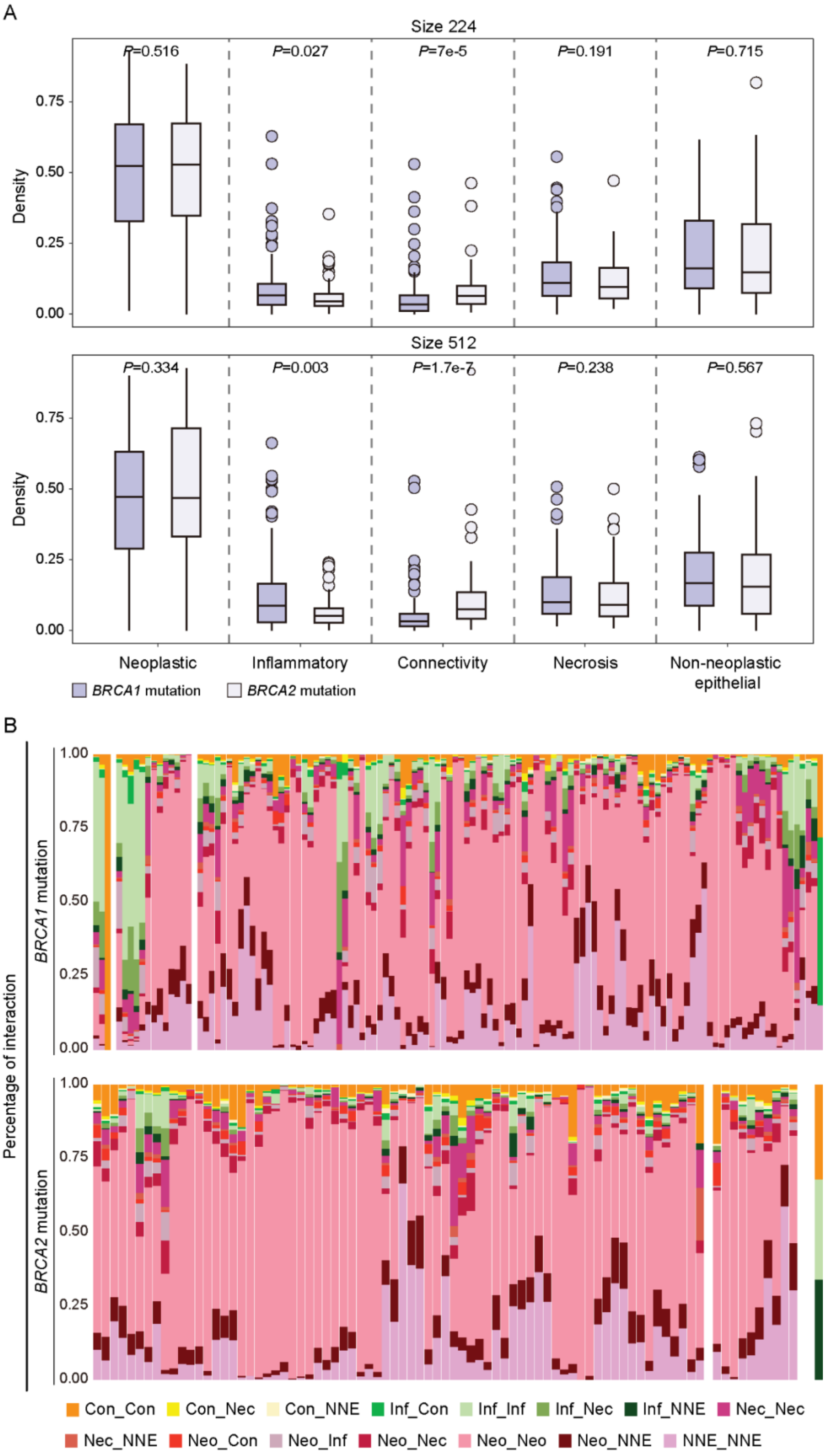
A
Size 224
P=0.516
P=0.027
P=7e-5
P=0.191
P=0.715
Size 512
P=0.334
P=0.003
P=1.7e-7
P=0.238
P=0.567
Density
0.00
0.25
0.50
0.75
Neoplastic
Inflammatory
Connectivity
Necrosis
Non-neoplastic epithelial
BRCA1 mutation
BRCA2 mutation
B
Percentage of interaction
BRCA1 mutation
BRCA2 mutation
0.00
0.25
0.50
0.75
1.00
Con_Con
Con_Nec
Con_NNE
Inf_Con
Inf_Inf
Inf_Nec
Inf_NNE
Nec_Nec
Nec_NNE
Neo_Con
Neo_Inf
Neo_Nec
Neo_Neo
Neo_NNE
NNE_NNE

Figure S5. Cell density and spatial cell-cell interaction of *BRCA1* and *BRCA2* mutation carriers. (**A**) Densities of cells in *BRCA1* and *BRCA2* mutation carriers. (**B**) Distribution of cell-cell interactions in *BRCA1* and *BRCA2* mutation carriers.

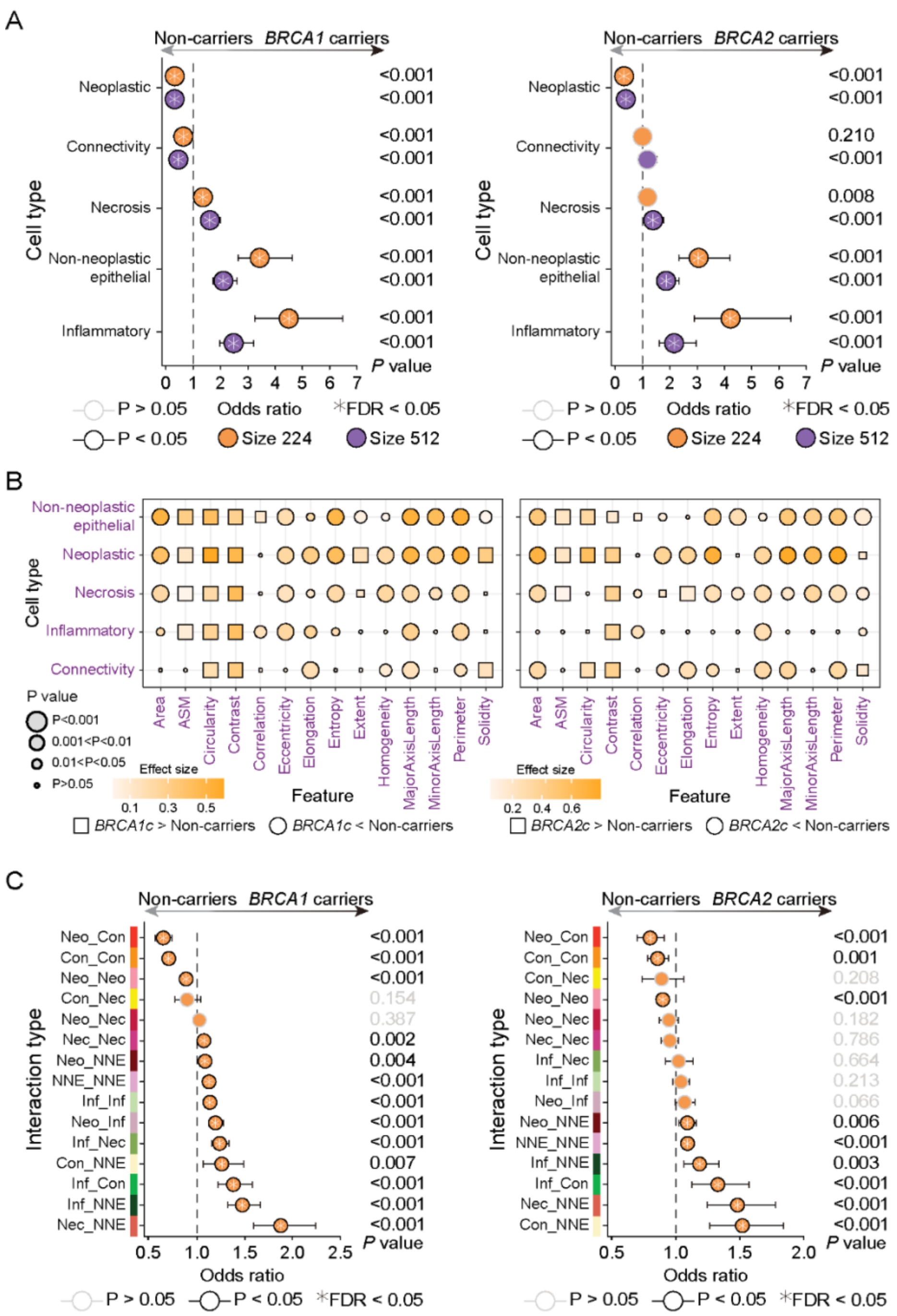

Figure S6. Characterization of cellular morphological and spatial interaction features between non-carriers and *BRCA1* and *BRCA2* mutation carriers. (**A**) Odds ratio for associations between cell densities and *BRCA1* and *BRCA2* mutation status. (**B**) Geometric and texture features of cells in *BRCA1* and *BRCA2* mutation carriers. (**C**) Odds ratio for associations between cell-cell interactions and *BRCA1* and *BRCA2* mutation status.

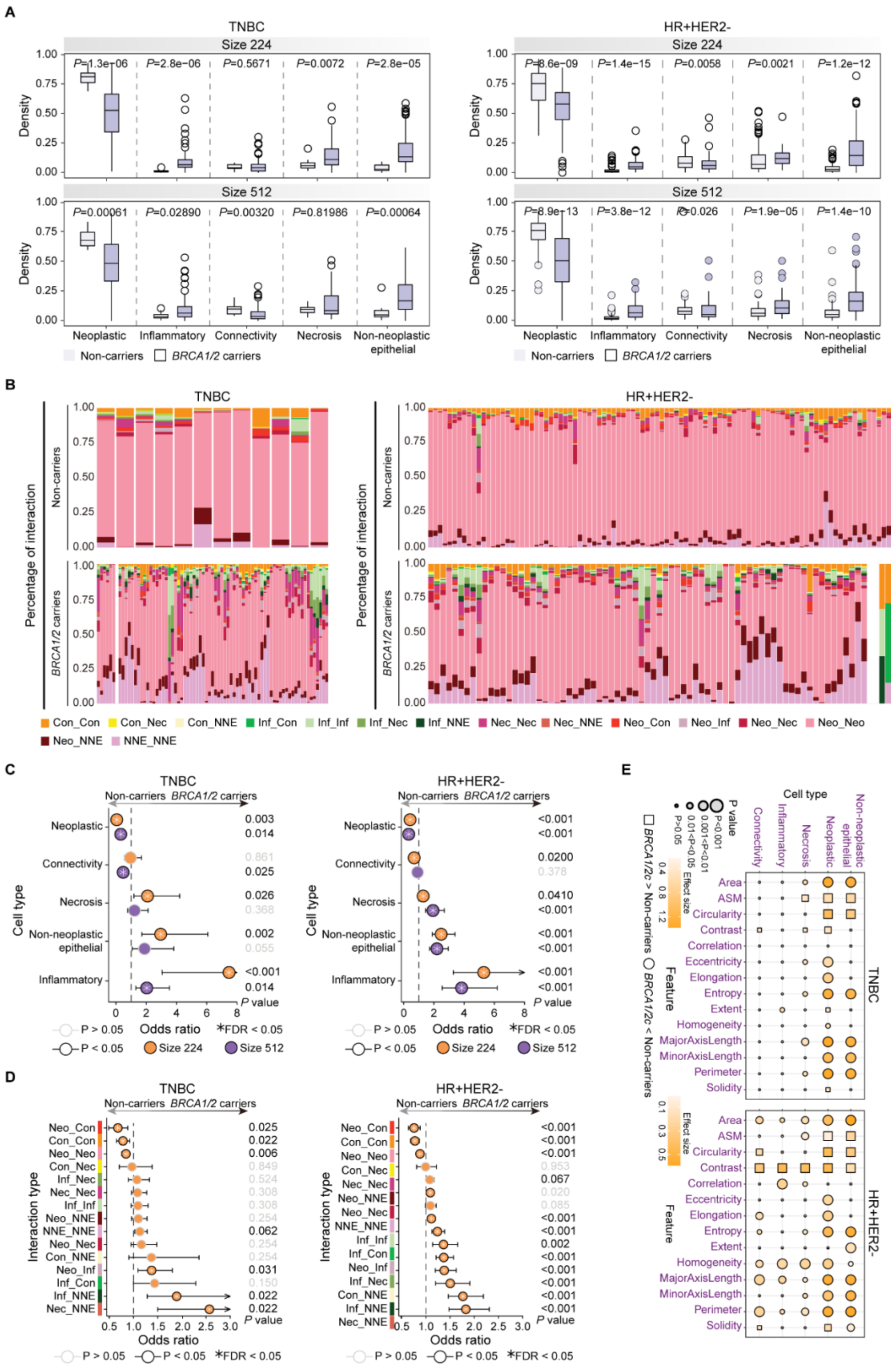
A
TNBC
Size 224
P=1.3e−06
P=2.8e−06
P=0.5671
P=0.0072
P=2.8e−05
Size 512
P=0.00061
P=0.02890
P=0.00320
P=0.81986
P=0.00064
Density
1.00
0.75
0.50
0.25
0.00
Neoplastic
Inflammatory
Connectivity
Necrosis
Non-neoplastic epithelial
Non-carriers
BRCA1/2 carriers
HR+HER2-
Size 224
P=8.6e−09
P=1.4e−15
P=0.0058
P=0.0021
P=1.2e−12
Size 512
P=8.9e−13
P=3.8e−12
P=0.026
P=1.9e−05
P=1.4e−10
B
TNBC
HR+HER2-
Percentage of interaction
Non-carriers
BRCA1/2 carriers
Con_Con
Con_Nec
Con_NNE
Inf_Con
Inf_Inf
Inf_Nec
Inf_NNE
Nec_Nec
Nec_NNE
Neo_Con
Neo_Inf
Neo_Nec
Neo_Neo
Neo_NNE
NNE_NNE
C
TNBC
Non-carriers
BRCA1/2 carriers
Cell type
Neoplastic
Connectivity
Necrosis
Non-neoplastic epithelial
Inflammatory
0.003
0.014
0.861
0.025
0.026
0.368
0.002
0.055
<0.001
0.014
P value
Odds ratio
P > 0.05
P < 0.05
*FDR < 0.05
Size 224
Size 512
HR+HER2-
<0.001
<0.001
0.0200
0.378
0.0410
<0.001
<0.001
<0.001
<0.001
<0.001
D
TNBC
Interaction type
Neo_Con
Con_Con
Neo_Neo
Con_Nec
Inf_Nec
Nec_Nec
Inf_Inf
Neo_NNE
NNE_NNE
Neo_Nec
Con_NNE
Neo_Inf
Inf_Con
Inf_NNE
Nec_NNE
0.025
0.022
0.006
0.849
0.524
0.308
0.308
0.254
0.062
0.254
0.254
0.031
0.150
0.022
0.022
HR+HER2-
Neo_Con
Con_Con
Neo_Neo
Con_Nec
Nec_Nec
Neo_NNE
Neo_Nec
NNE_NNE
Inf_Inf
Inf_Con
Neo_Inf
Inf_Nec
Con_NNE
Inf_NNE
Nec_NNE
<0.001
<0.001
<0.001
0.953
0.067
0.020
0.085
<0.001
<0.001
0.002
<0.001
<0.001
<0.001
<0.001
<0.001
E
Cell type
Connectivity
Inflammatory
Necrosis
Neoplastic
Non-neoplastic epithelial
P value
P<0.001
0.001<P<0.01
0.01<P<0.05
P>0.05
Effect size
BRCA1/2c > Non-carriers
BRCA1/2c < Non-carriers
Feature
Area
ASM
Circularity
Contrast
Correlation
Eccentricity
Elongation
Entropy
Extent
Homogeneity
MajorAxisLength
MinorAxisLength
Perimeter
Solidity
TNBC
HR+HER2-

Figure S7. Cell geometry and tumor microenvironment analysis in TNBC and HR+/HER2- breast cancer patients. (**A**) Densities of epithelial and TME cells of germline *BRCA1/2* mutation carriers and non-carriers in TNBC and HR+/HER2- patients. (**B**) High-scale distribution of cell-cell interactions of germline BRCA1/2 mutation carriers and non-carriers in TNBC and HR+/HER2- patients. (**C**) Odds ratios for associations between cell densities and germline BRCA1/2 mutation status in TNBC and HR+/HER2- patients. (**D**) Odds ratios for associations between cell-cell interactions and BRCA1/2 mutation status in TNBC and HR+/HER2- patients. (**E**) Low-scale geometric and textural features of cells of BRCA1/2 mutation carriers and non-carriers in TNBC and HR+/HER2- patients.

Abbreviations: TNBC, triple-negative breast cancer; HR, hormone receptor; HER2, human epidermal growth factor receptor 2.

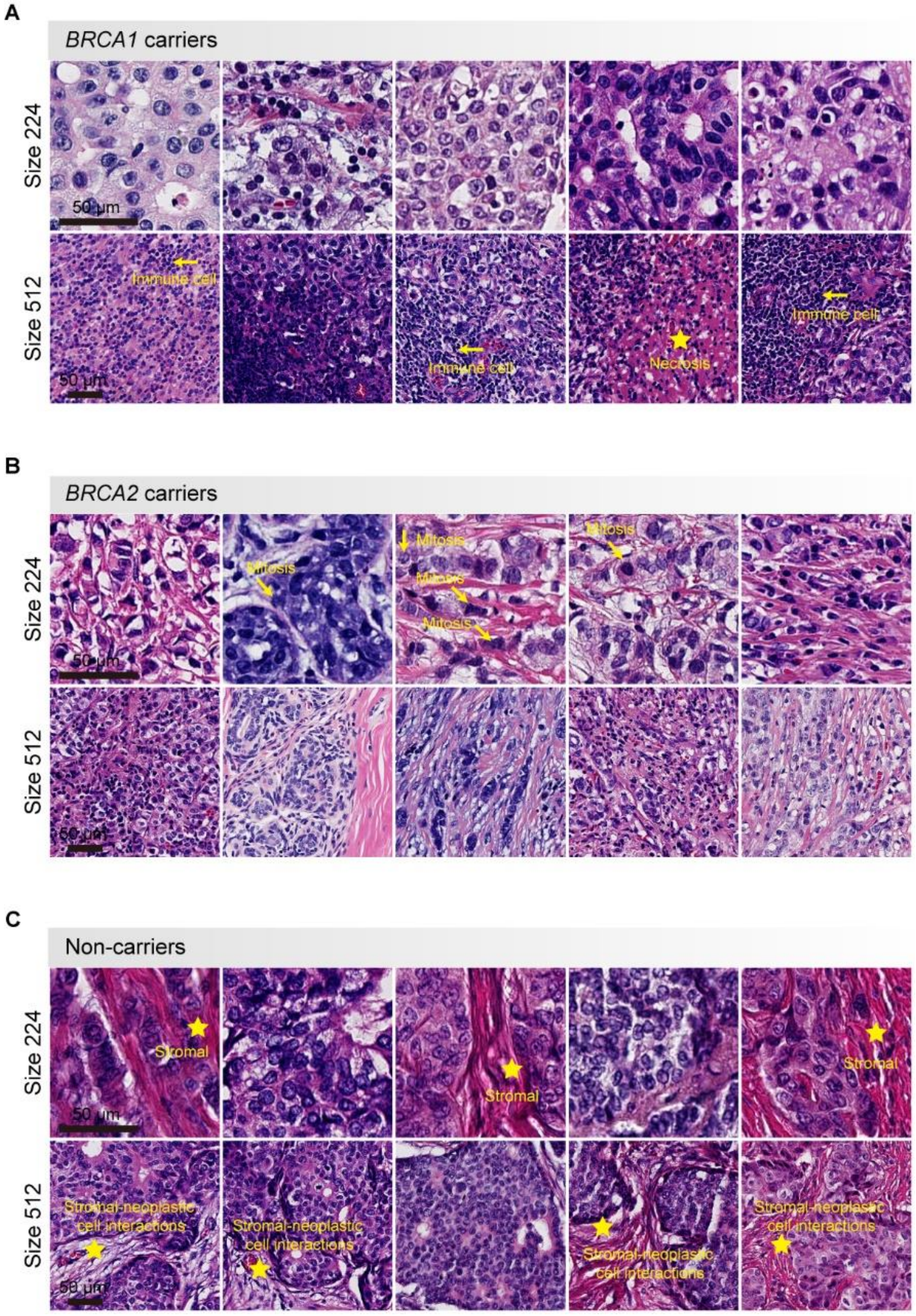

Figure S8. Representative patches reflecting these genotype-related tumor microenvironment features in germline *BRCA1*, *BRCA2* mutation carriers and non-carriers. Representative patches of *BRCA1* mutation carriers **(A)**, *BRCA2* mutation carriers **(B)** and non-carriers **(C)** identified by WISE-BRCA.

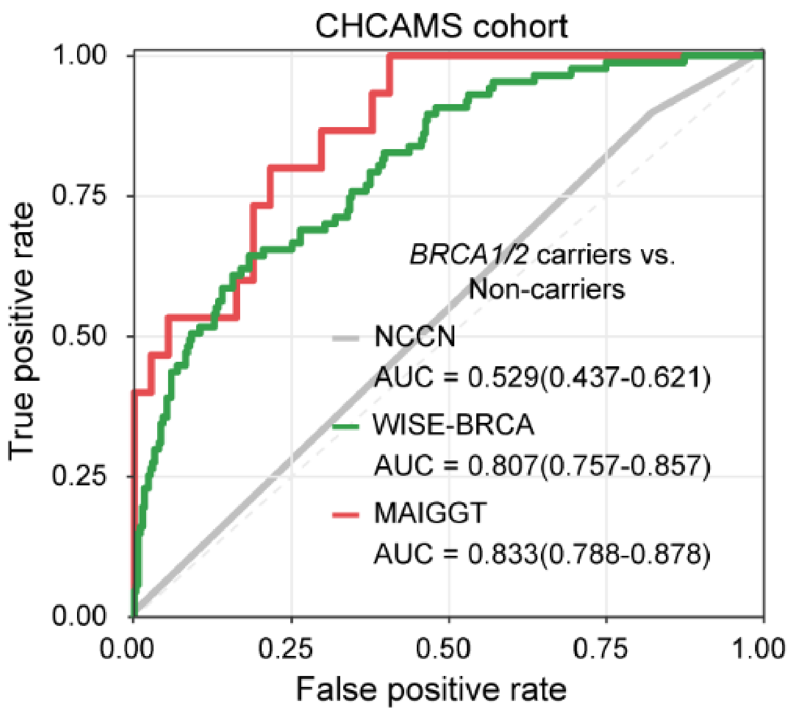

Figure S9. Prediction performance of MAIGGT in the CHCAMS cohort.

Abbreviations: MAIGGT: Multimodal Artificial Intelligence Germline Genetic Testing;

CHCAMS, Cancer Hospital, Chinese Academy of Medical Sciences.

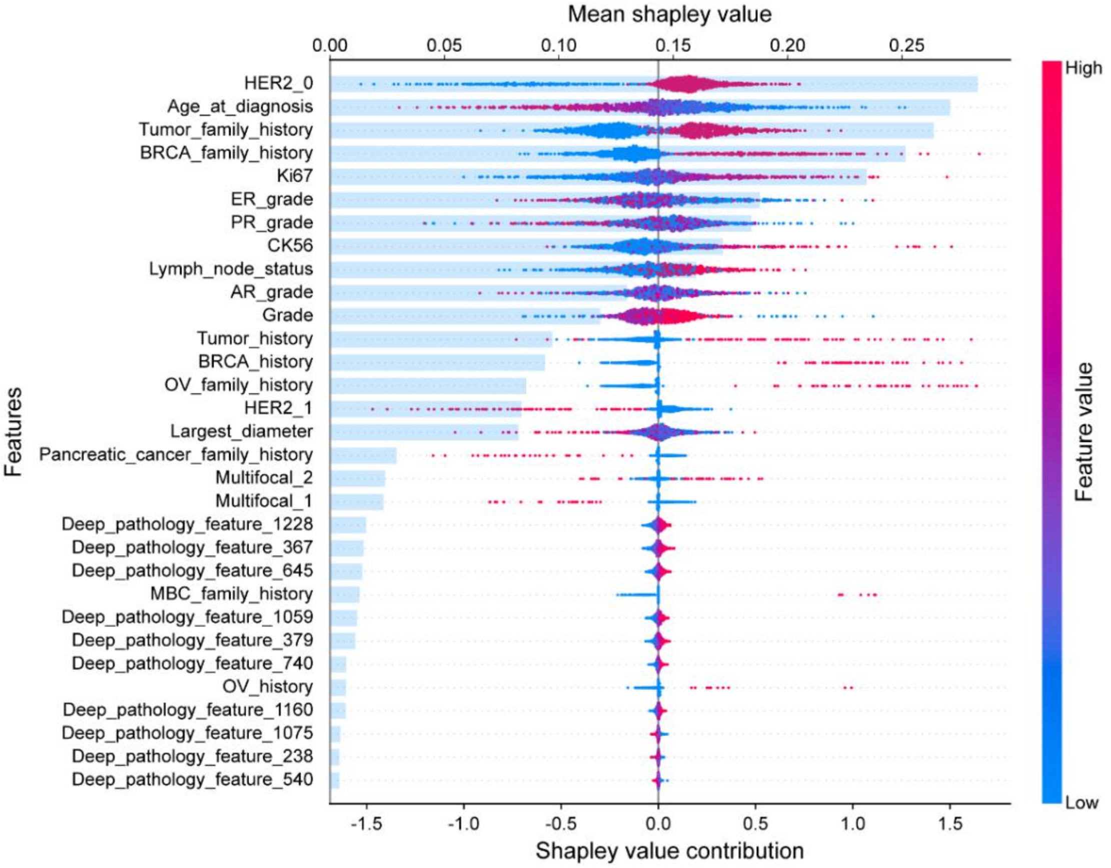

Figure S10. Interpretation by SHAP analysis for MAIGGT in germline BRCA1/2 mutation prediction.

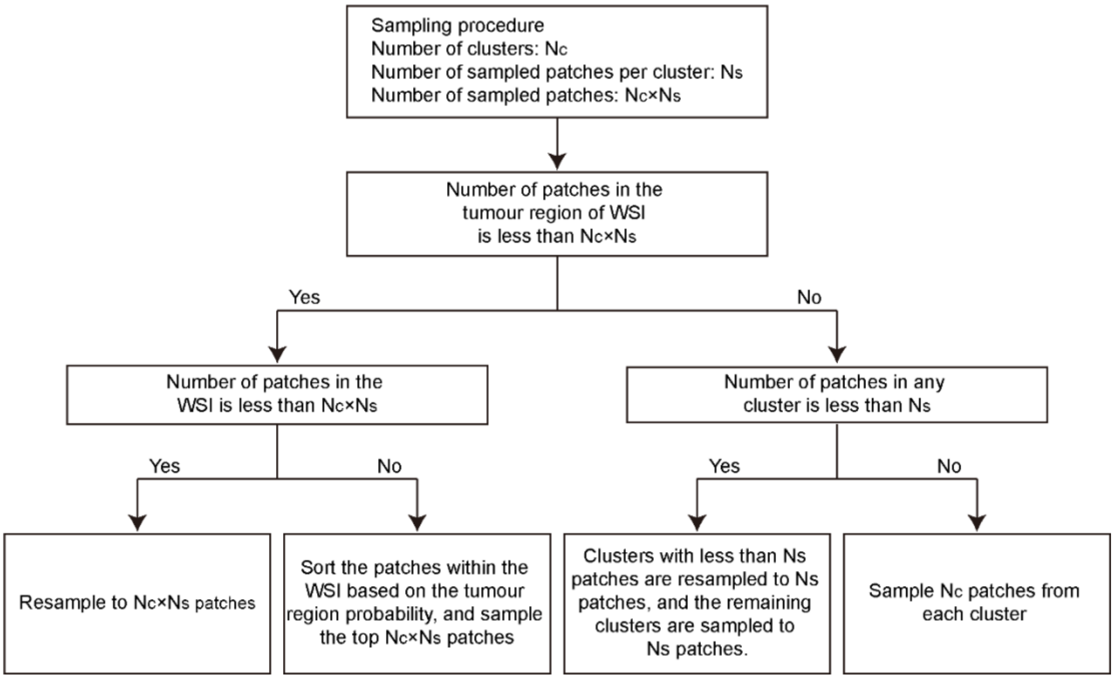

Figure S11. The process of clustering-based sampling method.

Abbreviations: WSI: whole slide image.

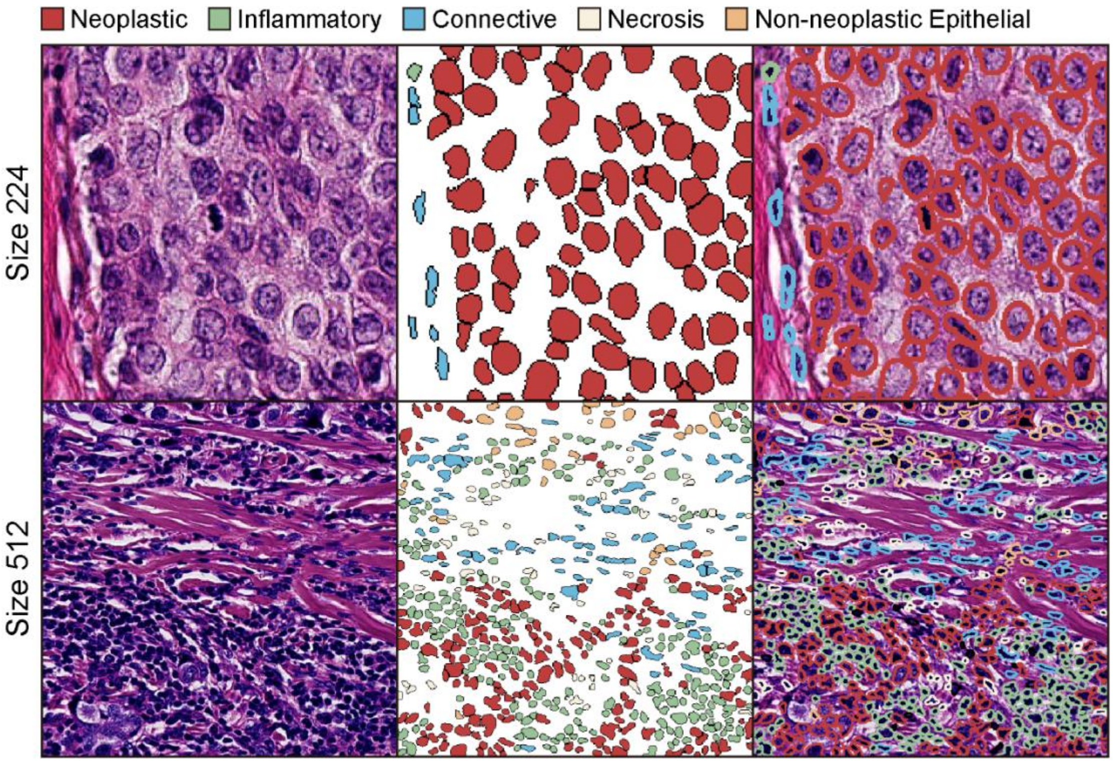

Figure S12. An example of cell segmentation using HoVer-Net on different sizes of patches from the CHCAMS cohort. The first column shows the original patch image, the second column displays the cell segmentation image of the patch, and the third column presents the overlay of the original patch image and the cell segmentation image.

Table S1. Comparison with benchmark methods based on different pathology foundation models.

Methods	Validation-SL	Validation-PL	Test-SL	Test-PL	YYH-SL	YYH-PL	HUMCH-SL	HMUCH-PL	Rank
ADMIL_CTransPath_224	0.491±0.014	0.471	0.547	0.558	0.606	0.57	0.678	0.591	52
ADMIL_Virchow2_224	0.788±0.02	0.835	0.732	0.764	0.687	0.585	0.75	0.764	9
ADMIL_H-Optimus-0_224	0.710±0.089	0.787	0.799	0.799	0.739	0.641	0.831	0.836	2
ADMIL_UNI_224	0.722±0.018	0.731	0.818	0.821	0.619	0.517	0.572	0.536	37
ADMIL_MUSK_224	0.713±0.009	0.731	0.752	0.746	0.715	0.702	0.646	0.691	24
ADMIL_CONCH_224	0.588±0.072	0.662	0.778	0.744	0.648	0.511	0.583	0.555	43
ADMIL_UNI_v2_224	0.749±0.015	0.768	0.809	0.823	0.714	0.61	0.685	0.655	16
ADMIL_CTransPath_512	0.581±0.044	0.645	0.651	0.649	0.749	0.674	0.738	0.709	36
ADMIL_Virchow2_512	0.814±0.012	0.863	0.721	0.754	0.699	0.659	0.764	0.736	5
ADMIL_H-Optimus-0_512	0.701±0.111	0.798	0.819	0.797	0.761	0.7	0.759	0.755	4
ADMIL_UNI_512	0.752±0.023	0.756	0.826	0.825	0.647	0.604	0.564	0.627	31
ADMIL_MUSK_512	0.751±0.014	0.763	0.769	0.74	0.759	0.719	0.647	0.6	17
ADMIL_CONCH_512	0.633±0.075	0.688	0.79	0.751	0.722	0.628	0.628	0.627	35
ADMIL_UNI_v2_512	0.772±0.005	0.769	0.802	0.814	0.709	0.658	0.67	0.673	12
GAMIL_CTransPath_224	0.582±0.071	0.641	0.589	0.599	0.537	0.636	0.247	0.164	56
GAMIL_Virchow2_224	0.540±0.008	0.54	0.568	0.599	0.55	0.343	0.799	0.8	49
GAMIL_H-Optimus-0_224	0.534±0.022	0.557	0.531	0.536	0.602	0.466	0.831	0.818	47
GAMIL_UNI_224	0.586±0.027	0.607	0.626	0.639	0.669	0.588	0.656	0.682	44
GAMIL_MUSK_224	0.528±0.038	0.571	0.514	0.475	0.551	0.513	0.853	0.873	46

GAMIL_CONCH_224	0.536±0.066	0.604	0.513	0.536	0.597	0.492	0.474	0.318	55
GAMIL_UNI_v2_224	0.586±0.027	0.607	0.626	0.639	0.669	0.588	0.656	0.682	44
GAMIL_CTransPath_512	0.591±0.069	0.66	0.585	0.609	0.569	0.671	0.429	0.409	51
GAMIL_Virchow2_512	0.732±0.078	0.819	0.76	0.765	0.694	0.609	0.773	0.764	7
GAMIL_H-Optimus-0_512	0.506±0.029	0.538	0.577	0.606	0.476	0.51	0.615	0.636	54
GAMIL_UNI_512	0.785±0.015	0.792	0.783	0.787	0.635	0.601	0.646	0.673	22
GAMIL_MUSK_512	0.535±0.039	0.57	0.552	0.542	0.512	0.466	0.722	0.809	50
GAMIL_CONCH_512	0.525±0.030	0.589	0.556	0.607	0.598	0.503	0.577	0.518	53
GAMIL_UNI_v2_512	0.544±0.020	0.56	0.567	0.54	0.649	0.486	0.766	0.727	48
CLAM_CTransPath_224	0.474±0.043	0.526	0.425	0.414	0.398	0.439	0.486	0.645	57
CLAM_Virchow2_224	0.729±0.092	0.836	0.739	0.732	0.686	0.648	0.569	0.582	33
CLAM_H-Optimus-0_224	0.758±0.017	0.801	0.823	0.841	0.625	0.483	0.829	0.827	6
CLAM_UNI_224	0.741±0.018	0.789	0.779	0.807	0.647	0.57	0.615	0.673	30
CLAM_MUSK_224	0.724±0.022	0.754	0.752	0.749	0.645	0.634	0.631	0.755	27
CLAM_CONCH_224	0.649±0.102	0.726	0.796	0.745	0.712	0.578	0.477	0.482	41
CLAM_UNI_v2_224	0.759±0.015	0.773	0.782	0.78	0.732	0.606	0.7	0.691	14
CLAM_CTransPath_512	0.616±0.107	0.706	0.736	0.735	0.689	0.675	0.678	0.673	34
CLAM_Virchow2_512	0.794±0.0210	0.842	0.737	0.732	0.697	0.667	0.639	0.627	19
CLAM_H-Optimus-0_512	0.732±0.078	0.819	0.76	0.765	0.694	0.609	0.773	0.764	7
CLAM_UNI_512	0.785±0.015	0.792	0.783	0.787	0.635	0.601	0.646	0.673	22
CLAM_MUSK_512	0.760±0.027	0.786	0.775	0.757	0.642	0.625	0.666	0.700	20
CLAM_CONCH_512	0.655±0.107	0.724	0.772	0.727	0.709	0.628	0.524	0.527	38

CLAM_UNI_v2_512	0.774±0.011	0.772	0.767	0.763	0.729	0.65	0.712	0.700	12
TransMIL_CTransPath_224	0.719±0.141	0.795	0.714	0.69	0.632	0.605	0.757	0.764	25
TransMIL_Virchow2_224	0.883±0.017	0.892	0.685	0.709	0.586	0.517	0.749	0.682	21
TransMIL_H-Optimus-0_224	0.864±0.038	0.919	0.657	0.614	0.704	0.613	0.887	0.855	3
TransMIL_UNI_224	0.758±0.170	0.865	0.718	0.709	0.562	0.473	0.871	0.945	10
TransMIL_MUSK_224	0.755±0.102	0.812	0.692	0.672	0.521	0.434	0.553	0.673	42
TransMIL_CONCH_224	0.696±0.084	0.799	0.755	0.741	0.661	0.513	0.55	0.518	39
TransMIL_UNI_v2_224	0.885±0.024	0.915	0.667	0.698	0.765	0.688	0.596	0.6	15
TransMIL_CTransPath_512	0.828±0.024	0.848	0.698	0.699	0.591	0.567	0.755	0.755	18
TransMIL_Virchow2_512	0.891±0.004	0.934	0.679	0.647	0.663	0.681	0.728	0.673	11
TransMIL_H-Optimus-0_512	0.795±0.201	0.897	0.663	0.622	0.66	0.484	0.767	0.745	28
TransMIL_UNI_512	0.802±0.154	0.878	0.663	0.643	0.653	0.572	0.697	0.764	26
TransMIL_MUSK_512	0.795±0.201	0.897	0.663	0.622	0.66	0.484	0.767	0.745	28
TransMIL_CONCH_512	0.713±0.116	0.816	0.744	0.715	0.621	0.508	0.588	0.518	40
TransMIL_UNI_v2_512	0.906±0.019	0.903	0.646	0.649	0.662	0.53	0.611	0.664	32
Proposed	0.754±0.014	0.789	0.807	0.824	0.800	0.798	0.783	0.800	1

Table S2. Clinical information from electronic medical records incorporated in MAIGGT.

Number	Parameters	Parameters type
1	Age at diagnosis	Age
2	History of any cancer	Personal cancer history
3	History of previous breast cancer	
4	History of ovarian cancer	
5	Bilateral breast cancer	
6	Family history of any cancer	Family cancer history
7	Family history of breast cancer	
8	Family history of ovarian cancer	
9	Family history of pancreas cancer	
10	Family history of male breast cancer	
11	The largest diameter of the tumor	Pathological features
12	Histological grade	
13	Multifocal breast cancer	
14	Androgen receptor status	
15	Estrogen receptor status	
16	Progesterone receptor status	
17	Ki67	
18	CK5/6_intensity	
19	Lymph nodes status	
20	HER2 positive	
21	HER2 negative	

Abbreviations: HER2: human epidermal growth factor receptor 2.

Table S3. Predictive performance under different fusion methods

	CHCAMS	YYH	HMUCH
	AUC	AUC	AUC
Softmax attention	0.753	0.570	0.871
Concatenation	0.732	0.642	0.721
Product	0.744	0.694	0.886
Plus	0.722	0.699	0.766
Proposed	0.833	0.845	0.925

Abbreviations: CHCAMS, Cancer Hospital, Chinese Academy of Medical Sciences; YYH, Yantai Yuhuangding Hospital ; HMUCH, Harbin Medical University Clinical Hospitals.

Table S4. Description of the geometric and texture features for segmented nuclei.

Feature name	Description
Area	Number of pixels of the nuclear region
ASM (angular second moment)	The angular second moment of the co-occurrence matrix
Circularity	The measurement of the roundness of the nuclear region. The circularity is defined by the ratio of the 4 pi times the area over the square of the perimeter
Contrast	The sum of squares variances of the co-occurrence matrix
Correlation	The correlation texture measures the linear dependency of grey levels on those of neighboring pixels.
Eccentricity	Eccentricity of the ellipse that has the same second-moments as the nuclear region. The eccentricity is the ratio of the focal distance (distance between focal points) over the major axis length
Elongation	Ratio of the major axis length over the minor axis length
Entropy	Measure the degree of confusion and disorder
Extent	Ratio of pixels in the region to pixels in the total bounding box
Homogeneity	Similarity of cellular morphology
MajorAxisLength	The length of the major axis of the ellipse that has the same normalized second central moments as the nuclear region.
MinorAxisLength	The length of the minor axis of the ellipse that has the same normalized second central moments as the nuclear region.
Perimeter	The average length of the boundary line of tumor cells
Solidity	The ratio of pixels in the object to pixels of the convex hull image

Table S5. Network architectures of each encoder and decoder in the mcVAE

Modal	Encoder	Decoder
Pathology image	LinearLayer(1536, 3072),<br>LeakyReLU()<br>LinearLayer(3072, 1536),<br>LeakyReLU()<br>LinearLayer(1536, 768)	LinearLayer(768, 768),<br>LeakyReLU()<br>LinearLayer(768, 1536),<br>LeakyReLU()<br>LinearLayer(1536, 1536)
Clinical information	LinearLayer(21, 192),<br>LeakyReLU()<br>LinearLayer(192, 384),<br>LeakyReLU()<br>LinearLayer(384, 768)	LinearLayer(768, 384),<br>LeakyReLU()<br>LinearLayer(384, 192),<br>LeakyReLU()<br>LinearLayer(192, 21)

Table S6. Network architectures of the multimodal fusion classification head

Layer	Multimodal fusion classification head
Layer 1	LinearLayer(1536, 768),<br>Tanh(),<br>Dropout()
Layer 2	LinearLayer(768, 384),<br>Tanh(),<br>Dropout()
Layer 3	LinearLayer(384, 1)
